# Thioredoxin‐2 Alleviates Osteoporosis by Restraining FOXO3a Nuclear Translocation

**DOI:** 10.1002/advs.78304

**Published:** 2026-10-18

**Authors:** Chi Zhan, Zhaocai Zhou, Yue Ding, Rong Lian, Xinyu Li, Xintong Zhang, Jie Cao, Zhonghui Tang, Zhengmei Lin, Zhi Song, Feng He

**Affiliations:** ^1^ School of Medicine Guangzhou First People's Hospital The Second Affiliated Hospital South China University of Technology Guangzhou China; ^2^ Guangdong Provincial Key Laboratory of Stomatology Hospital of Stomatology Guanghua School of Stomatology Sun Yat‐sen University Guangzhou China; ^3^ Zhongshan School of Medicine Sun Yat‐sen University Guangzhou China

**Keywords:** FOXO3a, osteoporosis, targeted therapy, Thioredoxin‐2, TRAIL

## Abstract

Osteoporosis is a prevalent age‐ and disease‐associated skeletal disorder characterized by bone loss, remodeling imbalance, and vascular niche deterioration, yet how endothelial redox dysfunction drives this process remains unclear. Here, we identify mitochondrial thioredoxin‐2 (TRX2) as an endothelial safeguard against osteoporotic bone loss. TRX2 was markedly reduced in aging bone vascular endothelial cells, and endothelial Trx2 deficiency accelerated skeletal degeneration by suppressing osteogenic activity and enhancing osteoclastogenesis. Mechanistically, TRX2 directly bound FOXO3a through a redox‐sensitive Cys90–Cys31 disulfide interaction, retaining FOXO3a at the mitochondrial outer membrane and limiting its nuclear translocation. TRX2 loss or oxidative stress disrupted this complex, enabling FOXO3a‐dependent transcriptional activation of tumor necrosis factor–related apoptosis‐inducing ligand (TRAIL) and generating a pro‐osteoclastogenic vascular niche. TRAIL neutralization or endothelial TRX2 restoration attenuated ovariectomy‐induced osteoporosis by preserving angiogenic–osteogenic coupling. These findings establish the endothelial TRX2–FOXO3a–TRAIL axis as a vascular redox checkpoint and potential therapeutic target for osteoporosis.

## Graphical abstract

Endothelial TRX2 preserves bone homeostasis by retaining FOXO3a at mitochondria and limiting TRAIL production. Loss of TRX2 or oxidative stress releases FOXO3a for nuclear translocation, increasing endothelial TRAIL secretion and driving osteoclast formation and bone loss. Restoring endothelial TRX2 or neutralizing TRAIL protects type H vessels, restrains bone resorption, and alleviates osteoporosis.

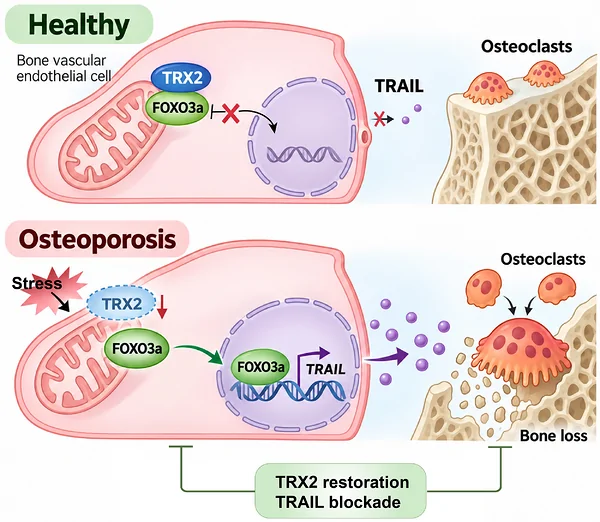

## 1 Introduction

Osteoporosis is a progressive systemic skeletal disorder characterized by diminished bone mass, microarchitectural deterioration, and increased fracture risk, with incidence rising sharply with age [1, 2, 3]. This osteoporosis burden is further amplified by chronic pathological conditions, including diabetes mellitus, chronic kidney disease, rheumatoid arthritis, and inflammatory bowel disease [4, 5, 6, 7]. Despite their diverse etiologies, these conditions converge on disrupted skeletal remodeling, in which osteoclast‐mediated bone resorption exceeds osteoblast‐mediated bone formation [8, 9, 10]. However, the mechanisms by which aging and chronic pathological conditions uncouple bone resorption from bone formation remain incompletely understood.

The skeleton is a highly vascularized organ in which blood vessels not only deliver oxygen, nutrients, and hematopoietic support but also provide instructive signals that regulate skeletal development, homeostasis, and repair [11, 12]. Bone vascular endothelial cells (BVECs) constitute a specialized vascular niche that coordinates osteoprogenitor differentiation and bone formation through angiogenesis–osteogenesis coupling [11, 13]. Among these vascular subtypes, type H endothelium, defined by high expression of CD31 and endomucin (CD31^hi^EMCN^hi^), is enriched in the metaphysis and endosteum, where it is closely associated with Osterix+ and RUNX2^+^ osteoprogenitors and couples vascular expansion to new bone formation [14, 15]. Degeneration of this vascular niche has been implicated in senile and pathological osteoporosis, partly through impaired skeletal perfusion, reduced endothelial Notch signaling, depletion of type H vessels, and subsequent suppression of bone formation [11, 16]. Nevertheless, how BVECs are transcriptionally remodeled during skeletal aging, and whether BVEC‐intrinsic dysfunction directly drives osteoporotic bone resorption and uncoupled skeletal remodeling, remain unresolved.

Oxidative stress and chronic inflammation are closely interconnected pathogenic processes that drive osteoporosis progression [17]. Aging, estrogen deficiency, hyperglycemia, and hyperlipidemia impair mitochondrial function and weaken antioxidant defenses, leading to excessive accumulation of reactive oxygen species (ROS) [18, 19]. Excess ROS disrupts redox‐sensitive pathways required for skeletal remodeling homeostasis and promotes sterile inflammation and cellular senescence [20]. Within the skeletal microenvironment, ROS‐induced senescence‐associated secretory programs increase pro‐inflammatory and osteoclastogenic mediators, including TNF‐α, IL‐1β, IL‐6, IL‐17, and receptor activator of NF‐κB ligand (RANKL), thereby enhancing osteoclast differentiation while suppressing osteoblast‐mediated bone formation [9, 17, 21, 22]. The clinical efficacy of denosumab, a monoclonal antibody targeting RANKL, underscores excessive osteoclastogenesis as a major therapeutic axis in osteoporosis [23, 24]. However, anti‐RANKL therapy primarily limits bone resorption and may not fully restore the osteogenic and vascular defects induced by oxidative stress and inflammation. Thus, although redox imbalance and inflammation‐driven osteoclastogenic signaling are recognized contributors to osteoporosis, the upstream endothelial mechanisms that link mitochondrial oxidative stress to inflammatory cytokine production and remodeling uncoupling remain poorly defined.

Thioredoxins are evolutionarily conserved redox proteins that regulate cellular oxidative status by catalyzing thiol‐disulfide exchange reactions [25]. Within this family, thioredoxin‐2 (TRX2) is a mitochondria‐localized antioxidant protein that limits oxidative stress, modulates inflammation, and delays cellular senescence [26]. Our previous study showed that adipocyte‐specific Trx2 deletion increased ROS production and activated NF‐κB‐associated mitophagy, thereby aggravating systemic inflammation and accelerating diabetic disease progression [27]. In the vascular system, TRX2 preserves endothelial function, promotes angiogenesis, and suppresses atherosclerosis by reducing oxidative stress, enhancing nitric oxide bioavailability, inhibiting ASK1 activity, and preventing oxidative stress‐induced apoptosis [28, 29]. Moreover, endothelial Trx2 loss impairs retinal vessel growth at early developmental stages and contributes to vascular malformation during aging [30]. In brief, these studies establish TRX2 as a key regulator of mitochondrial redox homeostasis and endothelial integrity. However, whether endothelial TRX2 preserves the skeletal vascular niche and whether its dysfunction directly contributes to osteoporotic remodeling imbalance remain unknown.

In this study, we sought to determine whether endothelial mitochondrial redox dysfunction directly contributes to osteoporosis by disrupting the skeletal vascular niche and remodeling homeostasis. Through single‐cell transcriptomic analysis, endothelial‐specific genetic models, mechanistic protein‐interaction assays, and therapeutic intervention studies, we identify endothelial TRX2 as a critical regulator of skeletal vascular integrity. We demonstrate that TRX2 decline in bone vascular endothelial cells promotes FOXO3a nuclear translocation and TRAIL‐dependent osteoclastogenesis, thereby linking endothelial redox imbalance to pathological bone resorption. Mechanistically, TRX2 restrains FOXO3a activity through a redox‐sensitive interaction that governs FOXO3a subcellular localization, defining an endothelial TRX2–FOXO3a–TRAIL axis that connects vascular dysfunction to osteoporotic bone loss. Therapeutic TRAIL blockade or endothelial TRX2 restoration mitigates osteoporotic remodeling imbalance, supporting this pathway as a potential intervention strategy for osteoporosis.

## 2 Results

### 2.1 Aging Remodels the Bone Vascular Endothelial Landscape

Aging‐associated osteoporosis is marked by progressive bone loss, with bone vascular endothelial cells (BVECs) emerging as key regulators of this process through their role in coupling angiogenesis to osteogenesis [1]. To define the transcriptomic dynamics of BVECs within the aging skeletal microenvironment, we interrogated a single‐cell RNA sequencing (scRNA‐seq) dataset of murine femoral endothelial cells spanning three chronological stages: juvenile (3 weeks), adult (12 weeks), and aged (75 weeks) [31]. Dimensionality reduction and unsupervised clustering resolved five transcriptionally distinct BVEC subpopulations (Figure 1A). Based on canonical marker expression, these were annotated as arterial ECs (aECs; *Sema3g, Gja4*, and *Bmx*), bone marrow ECs (bmECs; *Vcam1, Rgs4*, and *Stab2*), metaphyseal ECs (mpECs; *Piezo2, Ramp3*, and *Aplnr*), proliferating ECs (pECs; *Top2a, Neil3*, and *Mki67*), and remodeling ECs (rECs; *Cav1, Fmo2*, and *C1qtnf9*) (Figure 1B).

**FIGURE 1:**
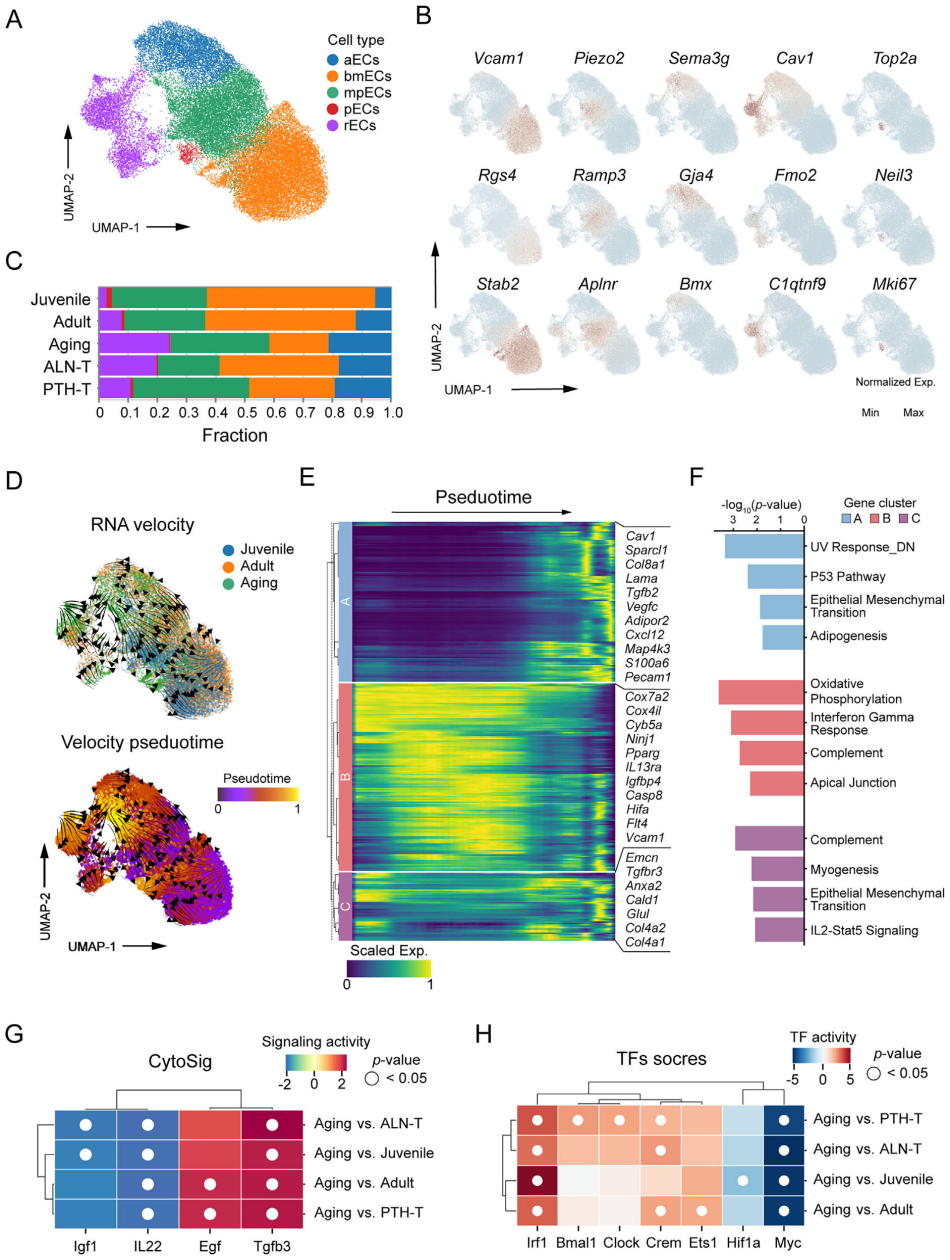
Single‐cell transcriptomics reveals aging‐associated remodeling of the bone vascular endothelial landscape. A) Uniform Manifold Approximation and Projection (UMAP) visualization of the endothelial cell (EC) compartment isolated from the femora of juvenile (3‐week‐old), adult (12‐week‐old), and aged (75‐week‐old) mice. ECs were resolved into five transcriptionally distinct subpopulations: aECs, bmECs, mpECs, pECs, and rECs. B) Feature plots showing the expression of canonical marker genes defining each EC subpopulation: aECs (*Sema3g, Gja4*, and *Bmx*), bmECs (*Vcam1, Rgs4*, and *Stab2*), mpECs (*Piezo2, Ramp3*, and *Aplnr*), pECs (*Top2a, Neil3*, and *Mki67*), and rECs (*Cav1, Fmo2*, and *C1qtnf9*). C) Stacked bar plots showing the relative proportions of EC subpopulations across juvenile, adult, aged, alendronate‐treated, and PTH‐treated groups. D) RNA velocity analysis of ECs from juvenile, adult, and aged mice, showing velocity fields (upper panels) and velocity pseudotime (lower panels) projected onto the UMAP embeddings. Arrows indicate the local average velocity calculated on a regular grid and represent projected future transcriptional states. E) Heatmap showing scaled expression of differentially expressed genes across the three pseudotime‐associated phases identified in (D). Genes were grouped into three major clusters according to their dynamic expression patterns, with representative genes indicated on the right. F) Gene Ontology (GO) enrichment analysis of differentially expressed genes from the three gene clusters shown in (E), highlighting phase‐specific biological pathways. G, H) Differential activation of (G) CytoSig‐inferred cytokine signaling signatures and (H) transcription factor activities in ECs from juvenile, adult, and aged mice. Circles indicate statistically significant differences between the indicated groups (*p* < 0.05).

Analysis of cellular composition revealed substantial age‐associated remodeling of the BVEC compartment. In aged mice, bmECs, which represent the specialized sinusoidal (type L) endothelium [32], were substantially depleted compared with juvenile and adult mice. By contrast, the proportion of mpECs, which correspond to type H endothelium [32], remained relatively stable across age groups (Figure 1C). These findings indicate that aging does not uniformly affect all endothelial subsets, but selectively disrupts distinct vascular niches within bone.

We next examined the temporal transcriptional programs associated with this age‐dependent endothelial remodeling. RNA velocity and pseudotime trajectory analyses revealed a directional continuum from juvenile to adult and ultimately to aged endothelial states (Figure 1D). Genes up‐regulated along the transition toward the aged state (Cluster A) were enriched for pathways associated with adipogenesis, epithelial‐mesenchymal transition, and p53 signaling (Figure 1E,F), consistent with features of cellular senescence and age‐related dysfunction. In contrast, genes preferentially expressed in juvenile or adult states, or shared across multiple stages (Clusters B and C), were mainly associated with metabolic, structural, and immune‐related processes (Figure 1E,F), suggesting a progressive loss of endothelial homeostatic programs during aging.

To further characterize functional changes in aged BVECs, we inferred cytokine signaling activities using CytoSig. Compared with juvenile BVECs, aged BVECs showed increased EGF and TGFβ3 signaling, whereas IGF1 and IL‐22 signaling were substantially diminished (Figure 1G). These changes suggest activation of tissue‐remodeling and profibrotic signaling programs, accompanied by attenuation of signals that support endothelial and skeletal homeostasis. Consistently, transcription factor activity analysis showed increased activity of IRF1 (Figure 1H), a key regulator of interferon signaling, inflammatory responses, cell‐cycle arrest, and apoptosis [33]. In parallel, the activities of MYC and HIF1A, two key regulators of endothelial proliferation and angiogenesis, were reduced (Figure 1H).

Together, these data demonstrate that skeletal aging is accompanied by broad transcriptional and signaling reprogramming of BVECs, characterized by inflammatory activation, impaired proliferative potential, and disruption of endothelial niche functions.

### 2.2 TRX2 Is Markedly Depleted in Aging BVECs

We next sought to identify molecular regulators that may underlie age‐associated BVEC deterioration. Differential gene expression analysis comparing aged BVECs with juvenile and adult counterparts revealed a set of commonly down‐regulated genes that was significantly enriched for mitochondrial functions (Figure 2A,B). Pathways related to apoptosis and oxidative stress responses were also altered, suggesting that aged BVECs acquire impaired mitochondrial stress resilience and increased susceptibility to cell death.

**FIGURE 2:**
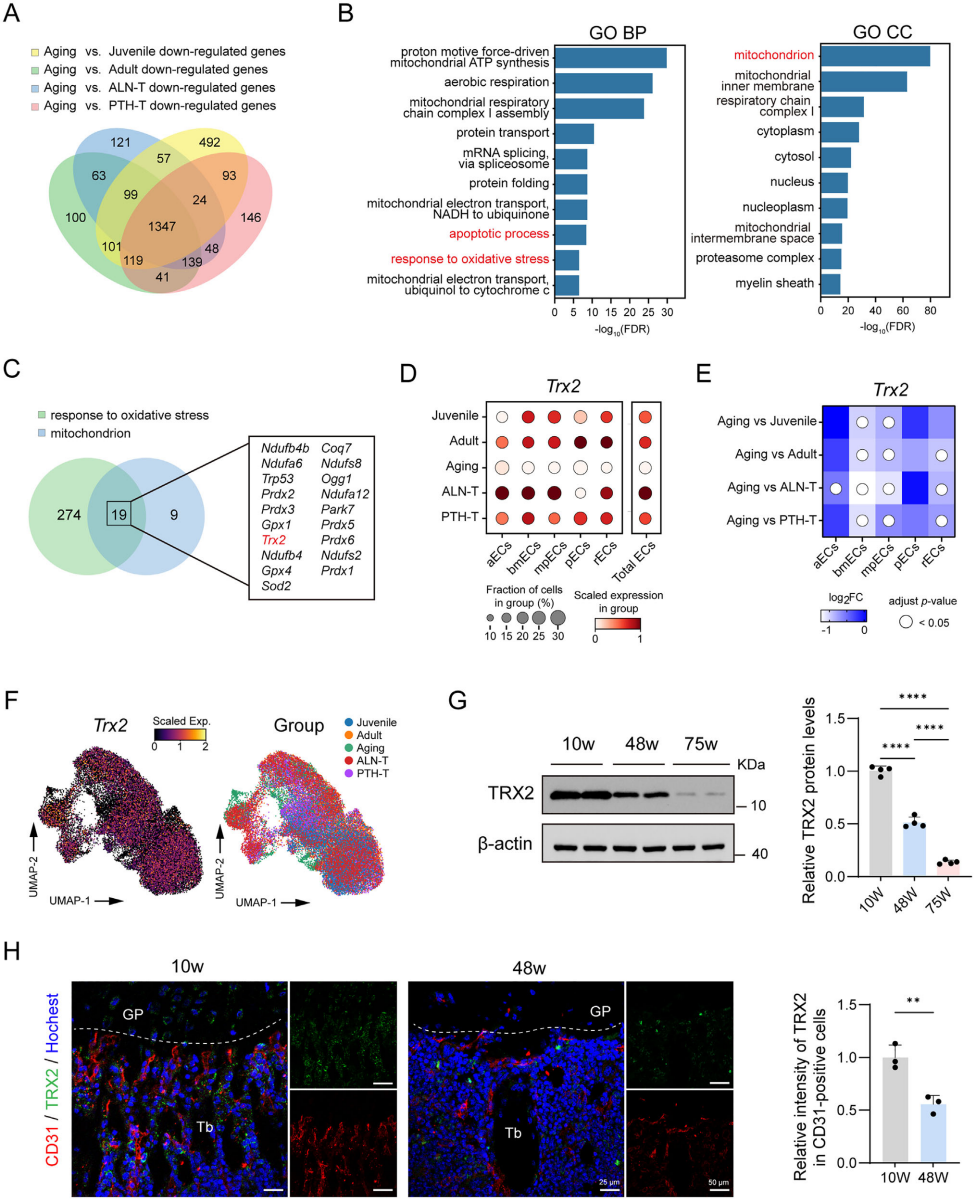
TRX2 is significantly down‐regulated in aging bone vascular endothelial cells (BVECs). A) Venn diagram showing the intersection of genes down‐regulated in aged ECs compared with juvenile, adult, ALN‐treated, and PTH‐treated ECs. B) Bar plots showing top Gene Ontology (GO) terms enriched among the intersecting down‐regulated genes in aged bone ECs. BP, biological process; CC, cellular component. C) Venn diagram showing the intersection between oxidative stress response‐related genes and mitochondria‐associated genes. D) Dot plot showing Trx2 expression across EC subclusters and age groups. Color indicates scaled expression, and dot size indicates the fraction of cells expressing Trx2 in each group. E) Heatmap showing TRX2 expression across EC subclusters in aged mice compared with the other groups. Color indicates scaled expression. White dots indicate statistically significant differences based on adjusted *p*‐value thresholds. F) UMAP plots showing TRX2 expression across age groups. G) Representative western blots and quantification of TRX2 protein levels in endothelial cells isolated from 10‐, 48‐, and 75‐week‐old mice (n = 4). β‐actin was used as a loading control. Relative protein levels were normalized to the 10‐week‐old group. H) Representative confocal images of TRX2 and CD31 immunofluorescence staining in femoral sections from 10‐ and 48‐week‐old mice (n = 3). GP, growth plate; Tb, trabecular bone. Scale bar, 25 µm. Quantification of TRX2 fluorescence intensity in femoral type H endothelial cells is shown. Source data for the unedited raw immunoblots shown in Figure S9. Data are presented as means ± SEM. Statistical significance was determined by one‐way ANOVA with Tukey's multiple‐comparisons test in (G) and unpaired Student's *t*‐test in (H). ^**^ *p* < 0.01, ^****^ *p* < 0.0001.

We therefore focused on genes co‐enriched in the categories of response to oxidative stress and mitochondria (Figure 2C). This analysis identified a conserved 19‐gene module, among which Trx2 emerged as a leading candidate. Trx2 encodes mitochondrial thioredoxin‐2, a key antioxidant regulator implicated in redox balance, inflammation, and cellular senescence [26, 34]. Notably, Trx2 was the most significantly down‐regulated gene in aged BVECs relative to all other age groups (Figure 2D–F). This decline was particularly pronounced in bmECs and mpECs (Figure 2E), two endothelial subsets closely associated with bone formation [11], suggesting that loss of TRX2 may preferentially affect osteogenesis‐supportive vascular niches.

We next validated the age‐dependent reduction of TRX2 at the protein level. Consistent with the transcriptomic data, TRX2 protein abundance progressively declined in BVECs from juvenile to adult and aged mice (Figure 2G). Immunofluorescence analysis of the metaphyseal region further confirmed this pattern in vivo. Compared with 10‐week‐old control mice, 48‐week‐old mice exhibited typical morphological characteristics of senile osteoporosis, with a significant decrease in CD31^+^ bone vessels. Spatial quantification revealed an approximately 50% reduction in TRX2 protein expression specifically within CD31^+^ BVECs in aged mice (Figure 2H).

These data identify endothelial TRX2 depletion as a prominent feature of the aging bone vascular niche and suggest that loss of TRX2 may contribute to the pathogenesis of age‐related osteoporosis.

### 2.3 Endothelial *Trx2* Deficiency Disrupts Bone Remodeling and Drives Osteoporotic Bone Loss

To determine whether endothelial TRX2 loss is sufficient to impair skeletal homeostasis, we generated endothelial cell‐specific *Trx2* conditional knockout mice (*Trx2^ECKO^*) by crossing *Trx2^floxed/floxed^* (*Trx2^fl/fl^*) mice with *Cdh5*‐Cre transgenic mice (Figure S1A). *Trx2^fl/fl^* mice with negative Cdh5‐Cre were used as controls (*Trx2^WT^*). Successful generation of *Trx2^ECKO^* mice was confirmed by PCR genotyping (Figure S1B) and further validated by a significant reduction of TRX2 protein in femur‐derived ECs from 3‐week‐old mutants (Figure S1C). Immunofluorescence staining further showed robust TRX2 expression in CD31^+^ BVECs from *Trx2^WT^* mice, whereas this signal was nearly abolished in *Trx2^ECKO^* mice (Figure 3A).

**FIGURE 3:**
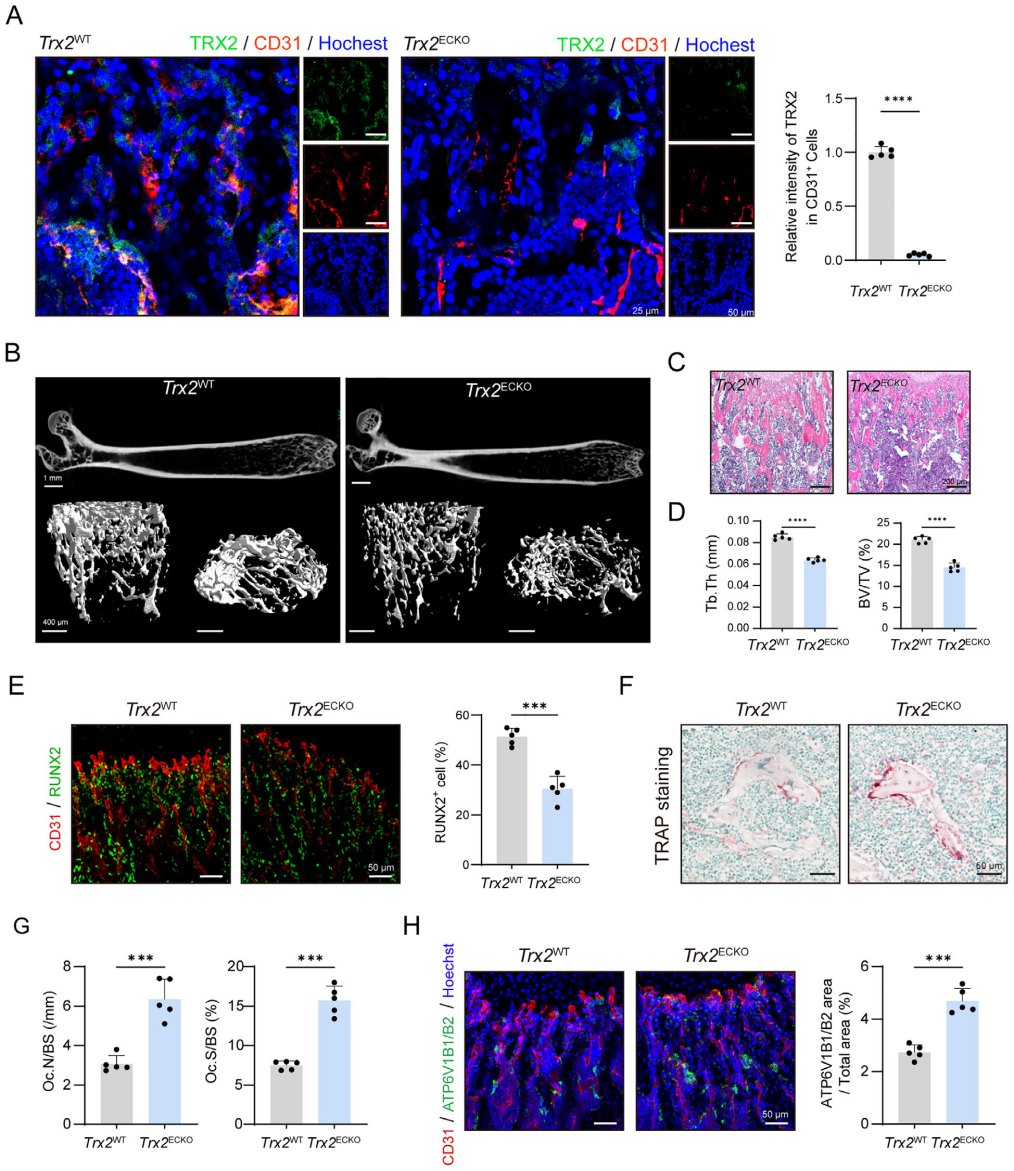
Endothelial TRX2 deficiency impairs bone remodeling and promotes osteoporotic bone loss. A) Representative immunofluorescence images of femoral bone sections from 10‐week‐old *Trx2* ^WT^ and *Trx2* ^ECKO^ mice stained for thioredoxin‐2 (TRX2; green), CD31 (red), and Hoechst (blue) (n = 5). Single‐channel images are shown on the right, and quantification shows reduced TRX2 fluorescence intensity in CD31^+^ endothelial cells. Scale bars, 25 µm. B) Representative micro–computed tomography (micro‐CT) images of femurs from *Trx2* ^WT^ and *Trx2* ^ECKO^ mice, including longitudinal sections and three‐dimensional reconstructions of trabecular bone in the distal femoral metaphysis (n = 5). Scale bars, 400 µm. C, D) Representative hematoxylin and eosin (H&E)–stained femoral sections (C) and micro‐CT‐based quantification of trabecular bone parameters (D), including trabecular thickness (Tb.Th) and bone volume/tissue volume (BV/TV). Scale bars, 200 µm. E) Representative immunofluorescence images and quantification of RUNX2^+^ osteoblast progenitors (green) and CD31^+^ vessels (red) in the metaphysis of *Trx2* ^WT^ and *Trx2* ^ECKO^ mice (n = 5). Scale bars, 25 µm. F, G) Representative tartrate‐resistant acid phosphatase (TRAP) staining images (F) and histomorphometric quantification (G) of osteoclast number per bone surface (Oc.N/BS) and osteoclast surface per bone surface (Oc.S/BS) in the metaphyseal region. Scale bars, 50 µm. H) Representative immunofluorescence images and quantification of ATP6V1B1/B2^+^ mature osteoclasts (green) and CD31^+^ vessels (red) in femoral bone sections (n = 5). Scale bars, 50 µm. All quantitative data are presented as means ± SEM. Statistical significance was determined by a two‐tailed unpaired Student's *t*‐test. ^***^ *p* < 0.001, ^****^ *p* < 0.0001.

Microcomputed tomography (micro‐CT) reconstruction and hematoxylin and eosin staining revealed pronounced trabecular rarefaction in 10‐week‐old *Trx2* ^ECKO^ mice compared with *Trx2^WT^* controls (Figure 3B,C). Quantitative morphometric analysis showed that trabecular thickness was reduced by approximately 23%, whereas bone volume per tissue volume decreased by approximately 31% in *Trx2* ^ECKO^ mice (Figure 3D). Notably, reductions in bone mass and structural integrity were already evident in 6‐week‐old *Trx2* ^ECKO^ mice (Figure S1D,E). These data establish that endothelial TRX2 is required to maintain trabecular bone architecture and that its loss is sufficient to induce early osteoporotic bone deterioration.

Given the essential role of the bone vasculature in coupling angiogenesis and osteogenesis, we next assessed osteogenic activity in the metaphyseal region. Immunofluorescence staining for RUNX2 and CD31 revealed a marked reduction in RUNX2^+^ osteoprogenitors in *Trx2* ^ECKO^ mice, with the RUNX2^+^ cell fraction decreased by 40% relative to control mice (Figure 3E). In contrast, indices of osteoclast activity were significantly increased. TRAP‐based histological analysis showed that osteoclast number per bone surface increased by approximately 2.1‐fold, whereas osteoclast surface per bone surface increased by approximately 2.2‐fold in *Trx2* ^ECKO^ mice relative to *Trx2* ^WT^ controls (Figure 3F,G). Consistently, immunofluorescence staining for the osteoclast‐associated marker ATP6V1B1/B2 showed an approximately 1.6‐fold increase in ATP6V1B1/B2‐positive area in the metaphyseal region of *Trx2* ^ECKO^ mice (Figure 3H).

Accordingly, these data demonstrate that endothelial Trx2 deficiency shifts the bone remodeling balance toward impaired osteogenesis and excessive osteoclastic resorption. Thus, loss of endothelial TRX2 is sufficient to drive osteoporotic skeletal deterioration.

### 2.4 TRX2 Restrains FOXO3a Nuclear Translocation Through a Redox‐sensitive Cys90–Cys31 Interaction Interface

To define the molecular mechanism by which TRX2 maintains endothelial homeostasis within the skeletal microenvironment, we identified TRX2‐interacting proteins by immunoprecipitation followed by mass spectrometry. This analysis identified FOXO3a as a TRX2‐associated protein (Figure 4A). FOXO3a is a key regulator of redox homeostasis, bone metabolism, and the pathogenesis of osteoporosis [35, 36]. High‐resolution immunofluorescence imaging and 3D spatial reconstruction confirmed substantial colocalization of TRX2 and FOXO3a, predominantly confined within the mitochondrial network (Figure 4B).

**FIGURE 4:**
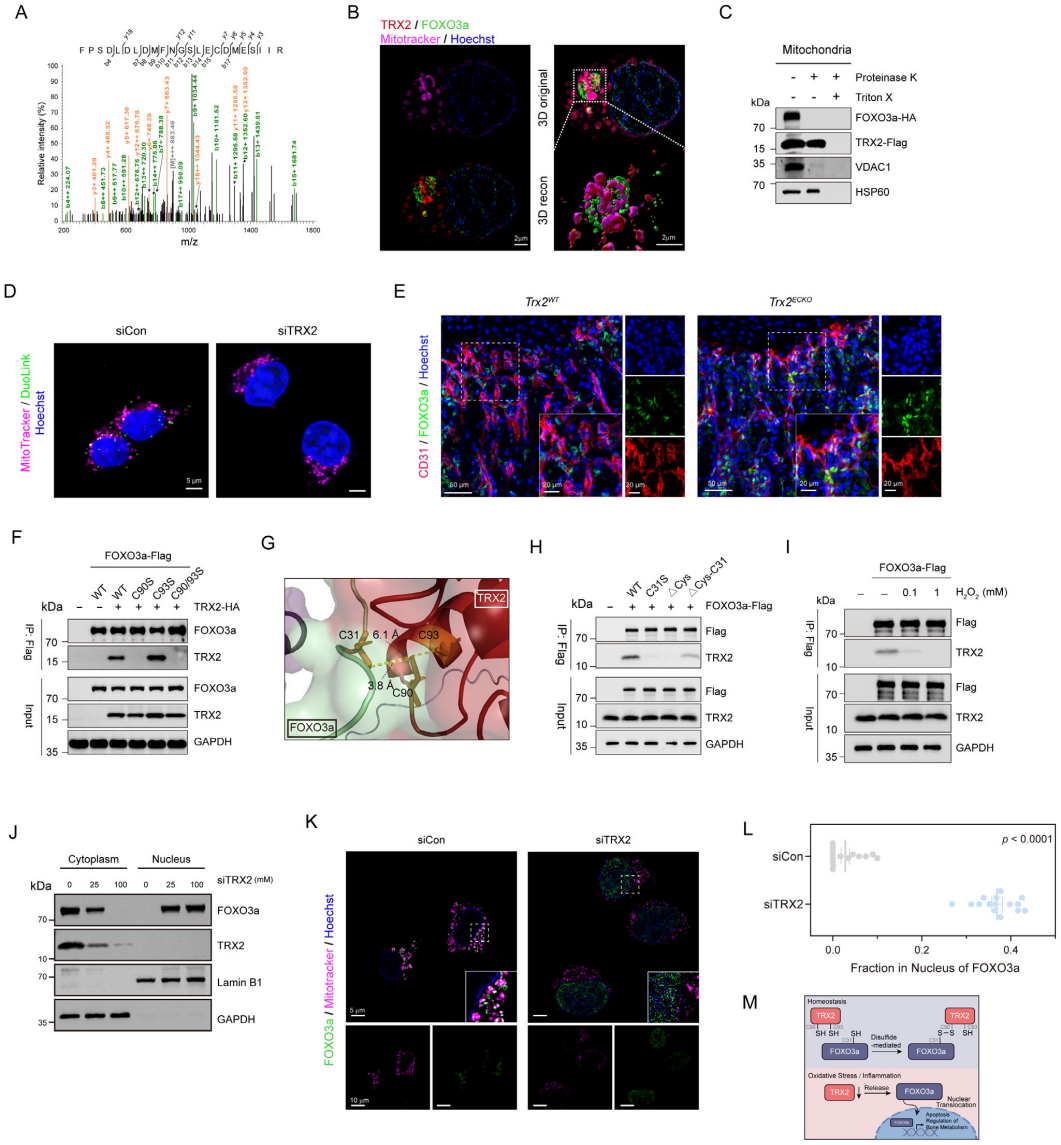
TRX2 interacts with FOXO3a through a redox‐sensitive Cys90–Cys31 interface and restrains FOXO3a nuclear translocation. A) Liquid chromatography–tandem mass spectrometry (LC‐MS/MS) analysis identifying FOXO3a as a TRX2‐binding partner. The representative MS/MS spectrum shows the peptide sequences of FOXO3a associated with TRX2. B) Structured illumination microscopy (SIM) images and 3D reconstructions showing colocalization of TRX2 (red) and FOXO3a (green) in HEK293T cells. Mitochondria and nuclei were labeled with MitoTracker (magenta) and Hoechst (blue), respectively. Scale bars, 2 µm. C) Proteinase K protection assay in isolated mitochondria treated with proteinase K in the presence or absence of Triton X‐100. VDAC1 and HSP60 served as mitochondrial outer membrane and matrix markers, respectively. D) Representative fluorescence images of HUVECs transfected with control siRNA (siCon) or TRX2‐targeting siRNA (siTRX2) and subjected to Duolink proximity ligation assay (PLA) using antibodies against TRX2 and FOXO3a. Green puncta represent PLA signals indicating close proximity between TRX2 and FOXO3a. Mitochondria were labeled with MitoTracker (magenta), and nuclei were counterstained with Hoechst (blue) (n = 3). Scale bars, 5 µm. E) Representative immunofluorescence images of distal femoral sections from 12‐week‐old *Trx2* ^WT^ and *Trx2* ^ECKO^ mice stained for CD31 (red), FOXO3a (green), and Hoechst (blue) (n = 3). Dashed boxes indicate the regions shown at higher magnification, with the corresponding single‐channel images presented on the right. Scale bars: 50 µm (overview images) and 20 µm (magnified and single‐channel images). F) Co‐immunoprecipitation (Co‐IP) assay in HEK293T cells transfected with FOXO3a‐Flag and different TRX2‐HA constructs, including wild‐type (WT), C90S, C93S, and C90/93S mutants, showing the requirement of TRX2 active‐site cysteines for FOXO3a binding. G) Molecular docking illustrating the predicted interface of the TRX2‐FOXO3a complex. The enlarged view shows the spatial proximity of TRX2 Cys90/Cys93 and FOXO3a Cys31. H) Co‐IP assay showing the interaction between endogenous TRX2 and FOXO3a‐Flag, including WT and cysteine‐mutant FOXO3a constructs. I) Co‐IP assay showing that the TRX2–FOXO3a interaction is sensitive to oxidative stress induced by hydrogen peroxide (H_2_O_2_). J, K) Western blot analysis of nucleocytoplasmic fractionation (J) and representative immunofluorescence images (K) showing the effect of siCon or siTRX2 treatment on FOXO3a subcellular distribution. Lamin B1 and GAPDH served as nuclear and cytosolic markers, respectively. Scale bars: 5 µm. L) Quantification of the nuclear fraction of FOXO3a corresponding to the immunofluorescence images in (K) (n = 5, with three technical replicates). M) Proposed model summarizing the mechanism by which TRX2 regulates FOXO3a subcellular localization. Source data for the unedited raw immunoblots shown in Figure S9. All quantitative data are presented as means ± SEM. Statistical significance was determined by a two‐tailed unpaired Student's *t*‐test. ^****^ *p* < 0.0001.

To elucidate the submitochondrial localization of this complex, we performed proteinase K protection assays using isolated mitochondria. In intact mitochondria, FOXO3a was highly sensitive to proteinase K digestion and was completely degraded. In contrast, TRX2 was partially reduced, whereas the mitochondrial matrix marker HSP60 remained largely protected in the absence of Triton X‐100. Upon membrane permeabilization with Triton X‐100, both TRX2 and HSP60 were degraded (Figure 4C). Consistently, Duolink proximity ligation assay (PLA) indicated discrete green puncta closely associated with MitoTracker‐labeled mitochondria in control HUVECs. These signals were nearly abolished following TRX2 knockdown, supporting the in situ proximity of TRX2 and FOXO3a at mitochondria (Figure 4D and Figure S2A). Importantly, in vivo immunofluorescence revealed pronounced FOXO3a accumulation within the Hoechst‐positive nuclei of CD31^+^ BVECs in *Trx2* ^ECKO^ mice, with nuclear FOXO3a fluorescence intensity increasing approximately 5.5‐fold relative to *Trx2* ^WT^ controls (Figure 4E and Figure S2B). These data suggest that FOXO3a associates with the mitochondrial surface, providing a spatial basis for its interaction with TRX2 at the mitochondrial outer membrane.

We next sought to map the interaction interface between TRX2 and FOXO3a. TRX2 contains two conserved redox‐active cysteine (Cys) residues, Cys90 and Cys93, within the catalytic center [37]. Co‐immunoprecipitation assays showed that wild‐type TRX2 was efficiently pulled down by FOXO3a. Mutation of TRX2 Cys90 to serine markedly disrupted FOXO3a binding, whereas the Cys93‐to‐serine mutant retained FOXO3a‐binding ability. Mutation of both Cys90 and Cys93 largely abolished the interaction with FOXO3a (Figure 4F). These data indicate that TRX2 Cys90, rather than Cys93, is the principal cysteine residue required for stable TRX2–FOXO3a complex formation.

To further investigate the structural basis of this interaction, we utilized AlphaFold3 to model the TRX2–FOXO3a complex. The predicted model achieved a DockQ score of 0.715, indicating a high‐confidence interaction well above the threshold for incorrect models (< 0.23). In this model, FOXO3a Cys31 was positioned closer to TRX2 Cys90 than to TRX2 Cys93, with predicted distances of 3.8 Å and 6.1 Å, respectively (Figure 4G). This structural prediction was consistent with the mutational data. Mutation of FOXO3a Cys31, or removal of the cysteine residues from FOXO3a, abolished TRX2 binding (Figure 4H). Conversely, reintroduction of Cys31 into the cysteine‐deficient FOXO3a mutant fully restored the interaction (Figure 4H). Moreover, exogenous H_2_O_2_ treatment disrupted TRX2–FOXO3a association in a dose‐dependent manner (Figure 4I). Collectively, these findings demonstrate that the TRX2–FOXO3a complex is stabilized by a specific disulfide‐mediated interaction between TRX2‐Cys90 and FOXO3a‐Cys31 and is sensitive to oxidative stress.

We next evaluated the functional consequences and molecular dynamics of this spatial conformation. TRX2 knockdown or overexpression did not alter the total protein abundance of FOXO3a (Figure S3A,B), suggesting that TRX2 regulates FOXO3a localization rather than protein stability. Subcellular fractionation showed that siRNA‐mediated TRX2 depletion reduced cytoplasmic FOXO3a abundance while promoting FOXO3a accumulation in the nuclear fraction in a dose‐dependent manner (Figure 4J). Confocal microscopy confirmed that FOXO3a was largely retained in punctate mitochondria‐associated structures under control conditions, whereas TRX2 knockdown induced pronounced FOXO3a nuclear redistribution and reduced mitochondrial colocalization (Figure 4K). Quantitatively, the nuclear fraction of FOXO3a increased approximately 5.6‐fold after TRX2 knockdown (Figure 4L).

In summary, these data demonstrate that TRX2 restrains FOXO3a nuclear translocation through a redox‐sensitive interaction mediated by TRX2 Cys90 and FOXO3a Cys31. Under oxidative stress or TRX2‐deficient conditions, disruption of this interaction releases FOXO3a from mitochondria‐associated sequestration and permits its nuclear accumulation, thereby linking endothelial redox dysfunction to transcriptional reprogramming (Figure 4M).

### 2.5 Loss of TRX2 Activates FOXO3a‐dependent TRAIL Signaling to Promote Osteoclastogenesis

Given that endothelial TRX2 deficiency promoted FOXO3a nuclear accumulation, we next sought to identify FOXO3a‐regulated downstream effectors that connect endothelial redox dysfunction to pathological bone resorption. By intersecting FOXO3a target genes with genes upregulated in aged endothelial cells across multiple conditions in the scRNA‐seq dataset (Figure 5A), we identified five candidate FOXO3a‐associated genes: *Sirt1, Foxo1, Bcl2l11, Tnfsf10*, and *Ar*. Among these, *Tnfsf10*, which encodes tumor necrosis factor‐related apoptosis‐inducing ligand (TRAIL), exhibited an approximately 4.3‐fold elevation and emerged as a strong candidate effector (Figure 5B and Figure S4A), given prior evidence that TRAIL can promote osteoclast differentiation and bone resorption [38, 39]. Corroborating this finding in a clinical context, we found that TRAIL expression was elevated by approximately 1.7‐fold in patients with osteoporosis compared with normal controls (Figure 5C), suggesting that TRAIL activation is associated with osteoporotic bone loss and may represent a downstream output of FOXO3a signaling in dysfunctional endothelial cells. Consistently, we analyzed FOXO3a CUT&Tag profiles in BVECs to determine whether FOXO3a directly regulates *Tnfsf10*. TRX2 deficiency significantly increased FOXO3a enrichment at the *Tnfsf10* promoter, where two putative binding sites were identified (Figure S5A,B). Targeted CUT&Tag‐qPCR assay showed an approximately 9.0‐fold increase in FOXO3a enrichment at site 2 in *Trx2* ^ECKO^ BVECs compared with *Trx2* ^WT^ controls (Figure S5C), supporting direct transcriptional regulation of *Tnfsf10* by FOXO3a.

**FIGURE 5:**
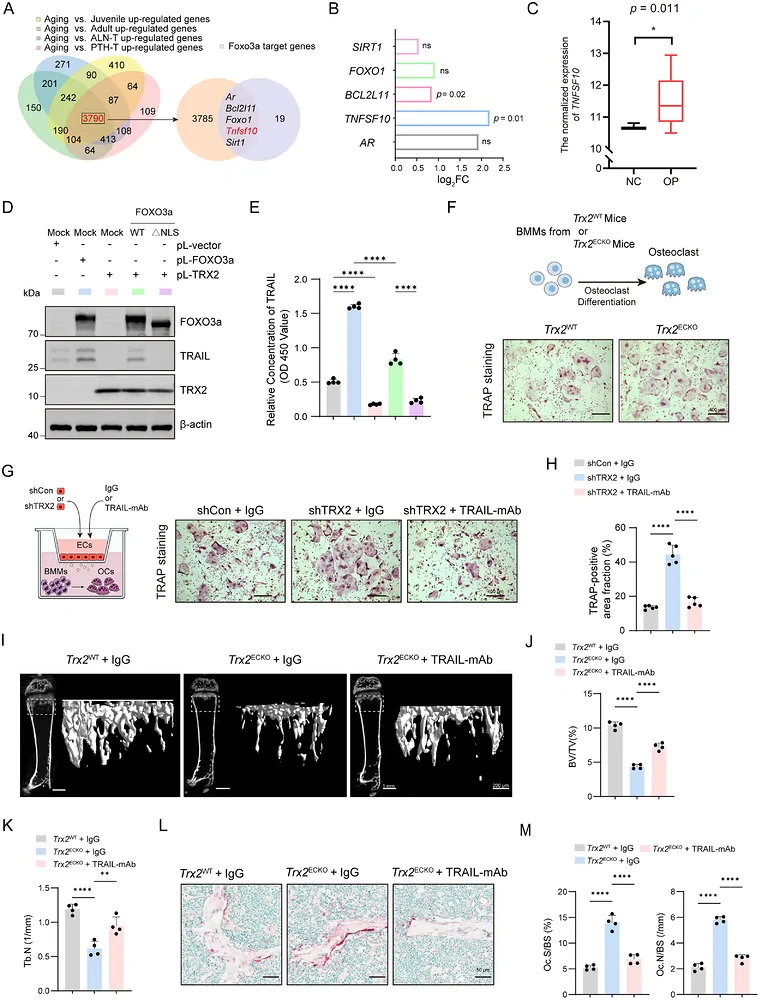
Endothelial TRX2 restrains osteoclastogenesis and osteoporotic bone loss through FOXO3a‐mediated TRAIL signaling. A) Venn diagram analysis identifying candidate downstream targets. Overlap between aging‐up‐regulated genes and FOXO3a target genes identified Tnfsf10, which encodes TNF‐related apoptosis‐inducing ligand (TRAIL), as a candidate mediator. B) Bar graph showing the log_2_ fold change of selected FOXO3a target genes in the GSE230665 dataset. C) Box plot showing normalized TRAIL expression in normal controls (NC) and patients with osteoporosis (OP) from the GSE230665 dataset. D) Western blot analysis of FOXO3a, TRAIL, and TRX2 protein levels in HEK293T cells transfected with pL‐vector, pL‐FOXO3a wild type (WT), pL‐FOXO3a ΔNLS mutant, or pL‐TRX2. β‐actin served as the loading control. E) Secreted TRAIL levels in the culture supernatants of the experimental groups in (D), as measured by enzyme‐linked immunosorbent assay (ELISA) (n = 4), with absorbance read at 450 nm. F) Representative TRAP staining images of osteoclasts derived from bone marrow‐derived macrophages (BMMs) isolated from *Trx2* ^WT^ and *Trx2* ^ECKO^ mice (n = 5). Scale bars, 400 µm. G, H) In vitro assessment of endothelial‐osteoclast crosstalk. (G) Left: schematic of the Transwell coculture system. Right: representative images of TRAP‐stained BMMs cocultured with control endothelial cells (shControl) or TRX2‐depleted endothelial cells (shTRX2) in the presence of immunoglobulin G (IgG) control or a TRAIL‐neutralizing monoclonal antibody (TRAIL‐mAb). Scale bars, 400 µm. (H) Quantification of the TRAP‐positive area fraction in (G) (n = 5). I‐K) In vivo rescue of TRX2 deficiency‐induced bone loss by TRAIL blockade. Representative 3D micro‐CT reconstructions of distal femurs from *Trx2* ^WT^ and *Trx2* ^ECKO^ mice treated with IgG or TRAIL‐mAb (I) (n = 4). Scale bars, 200 µm. Quantitative micro‐CT analysis of trabecular bone volume/tissue volume (BV/TV) (J) and trabecular number (Tb.N) (K). L, M) Histological evaluation of osteoclast formation in femoral sections. Representative images of TRAP‐stained femoral sections (L) and corresponding histomorphometric quantification of osteoclast surface per bone surface (Oc.S/BS) and osteoclast number per bone surface (Oc.N/BS) (M) (n = 4). Scale bars, 50 µm. Source data for the unedited raw immunoblots shown in Figure S10. Data in (C) are presented as means ± SEM, and statistical significance was determined by a two‐tailed unpaired Student's *t*‐test. All other quantitative data are presented as means ± SEM, with statistical significance determined by one‐way ANOVA with Tukey's multiple‐comparisons test. ^*^ *p* < 0.05, ^**^ *p* < 0.01, ^****^ *p* < 0.0001.

We next tested whether FOXO3a directly regulates TRAIL expression. FOXO3a overexpression markedly increased TRAIL expression, whereas disruption of FOXO3a nuclear localization (ΔNLS) attenuated this effect (Figure 5D). Moreover, TRX2 overexpression suppressed FOXO3a‐induced TRAIL production, as confirmed by ELISA analysis (Figure 5E), indicating that TRX2 limits TRAIL expression by restraining FOXO3a‐dependent transcriptional activity. To determine whether TRX2 deficiency promotes osteoclastogenesis, bone marrow macrophages (BMMs) were isolated from *Trx2* ^WT^ and *Trx2* ^ECKO^ mice and induced toward osteoclast differentiation. Functionally, BMMs derived from *Trx2* ^ECKO^ mice exhibited an enhanced osteoclastogenic capacity compared with those from *Trx2* ^WT^ mice, as evidenced by an approximately 3.5‐fold elevation in TRAP‐positive multinucleated cells after osteoclast induction (Figure 5F and Figure S4B). Furthermore, in an endothelial cell–osteoclast coculture system, TRX2 knockdown in endothelial cells markedly promoted osteoclastogenesis, resulting in an approximately 3.2‐fold increase in the TRAP‐positive area fraction. This effect was attenuated by TRAIL neutralization (Figure 5G,H). Importantly, FOXO3a silencing in *Trx2* ^ECKO^ BVECs reduced the elevated TRAIL expression caused by TRX2 deficiency (Figure S6A). In a BVECs‐osteoclast precursor transwell coculture system, FOXO3a silencing also attenuated the increase in the TRAP‐positive area fraction induced by *Trx2* ^ECKO^ BVECs (Figure S6B–D). Together, these findings identify the FOXO3a–TRAIL axis as a key mechanism by which endothelial TRX2 deficiency promotes osteoclastogenesis.

To confirm whether TRAIL contributes to bone loss in vivo, *Trx2* ^ECKO^ mice were treated with either control IgG or TRAIL‐neutralizing antibody, followed by micro‐CT and histological assessment. TRAIL blockade partially rescued trabecular bone loss in *Trx2* ^ECKO^ mice, as evidenced by increased bone volume per tissue volume and trabecular number compared with IgG‐treated controls (Figure 5I–K). Consistently, TRAP staining revealed a marked reduction in osteoclast number and surface following TRAIL neutralization treatment compared with IgG‐treated *Trx2* ^ECKO^ mice (Figure 5L,M), indicating suppressed osteoclast formation.

Thus, these data demonstrate that endothelial TRX2 deficiency drives osteoporotic bone loss by activating the FOXO3a–TRAIL signaling axis, thereby converting the bone vascular niche into a pro‐osteoclastogenic microenvironment, and accelerating pathological bone resorption.

### 2.6 TRAIL Blockade Protects Against Osteoporosis by Restoring Angiogenic–Osteogenic Coupling and Suppressing Osteoclastogenesis

Because TRAIL was identified as a downstream effector of FOXO3a in TRX2‐deficient endothelial cells, we next evaluated whether pharmacological TRAIL blockade could attenuate osteoporotic bone loss. We adopted the ovariectomy‐induced (OVX) osteoporosis model, which recapitulates the clinical pathogenesis of postmenopausal osteoporosis [1, 22]. Micro‐CT analysis revealed that ovariectomy induced severe deterioration of trabecular and cortical bone microarchitecture (Figure 6A,B). Systemic administration of a TRAIL‐neutralizing antibody substantially mitigated this pathological bone loss, increasing bone volume fraction and restoring trabecular number, trabecular thickness, cortical thickness, and bone mineral density relative to OVX mice treated with control IgG (Figure 6A,B). Histological examination via H&E staining further confirmed marked trabecular depletion and concurrent marrow adiposity in the osteoporotic OVX mice, both of which were attenuated by TRAIL blockade (Figure 6C).

**FIGURE 6:**
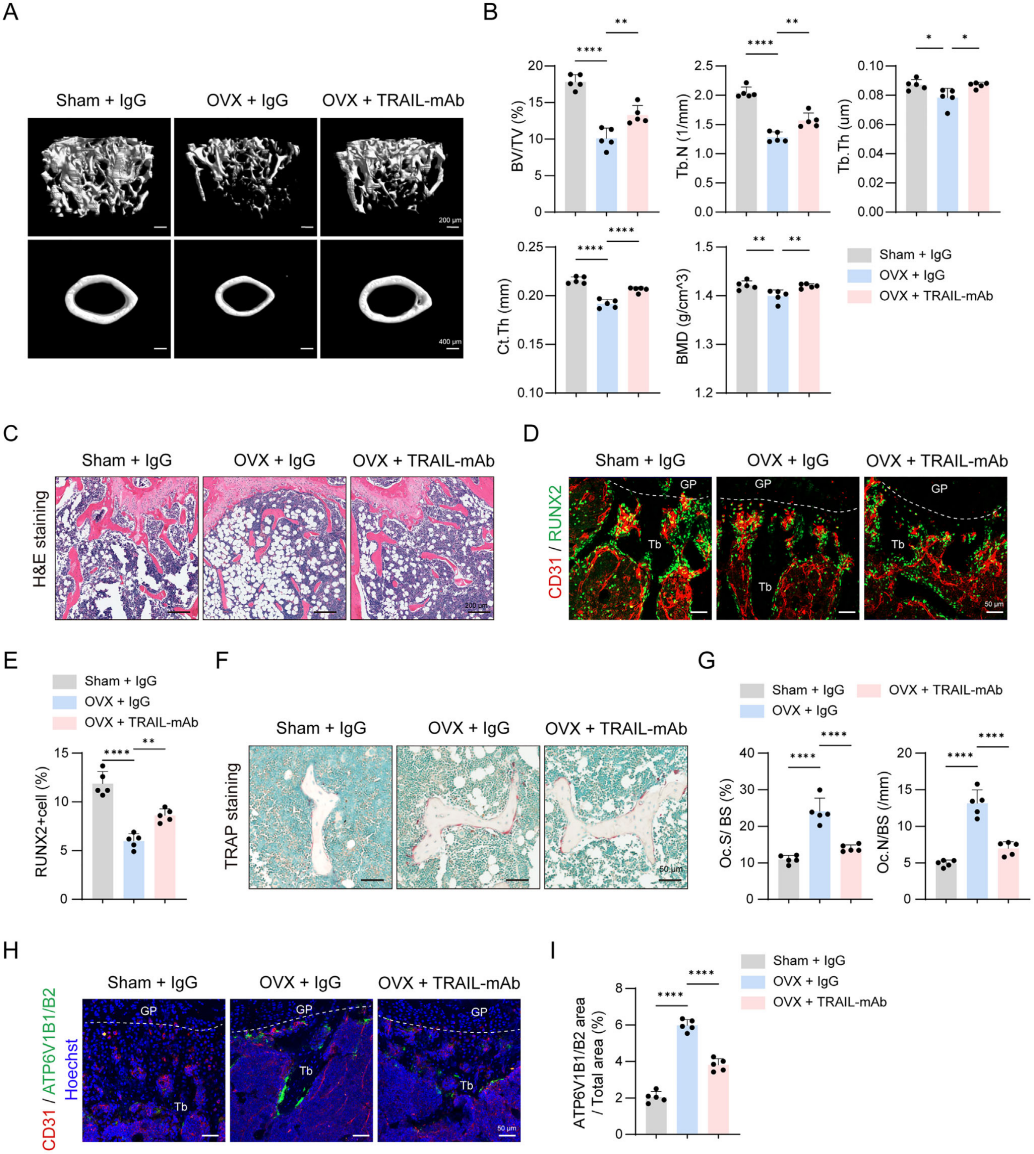
TRAIL blockade restores angiogenesis–osteogenesis coupling and attenuates OVX‐induced osteoporotic bone loss. A) Representative 3D micro‐CT reconstructions of trabecular bone (top) and cortical bone (bottom) in distal femurs from mice in the Sham +IgG, ovariectomy (OVX) + IgG, and OVX + TRAIL Ab groups (n = 5). Scale bars, 200 µm (top) and 400 µm (bottom). B) Quantitative micro‐CT analysis of trabecular bone volume/tissue volume (BV/TV), trabecular number (Tb.N), trabecular thickness (Tb.Th), cortical thickness (Ct.Th), and bone mineral density (BMD) in the indicated groups. C) Representative H&E–stained distal femoral sections showing trabecular architecture and bone marrow adiposity. Scale bars, 200 µm. D, E) Representative immunofluorescence images of CD31^+^ vessels (red) and RUNX2^+^ osteoblast‐lineage cells (green) in the femoral metaphysis (D), with corresponding quantification of the percentage of RUNX2^+^ cells (E). GP, growth plate; Tb, trabecular bone. Scale bars, 50 µm. F, G) Representative TRAP–stained distal femoral sections (F) and histomorphometric quantification of osteoclast surface per bone surface (Oc.S/BS) and osteoclast number per bone surface (Oc.N/BS) (G). Scale bars, 50 µm. H, I) Representative immunofluorescence images of CD31^+^ vessels (red) and ATP6V1B1/B2^+^ mature osteoclasts (green) in the femoral metaphysis (H), with corresponding quantification of the ATP6V1B1/B2^+^ area relative to the total area (I). GP, growth plate; Tb, trabecular bone. Scale bars, 50 µm. All quantitative data are presented as means ± SEM. Statistical significance was determined by one‐way analysis of variance (ANOVA) with Tukey's multiple‐comparisons test. ^*^ *p* < 0.05, ^**^ *p* < 0.01, ^****^ *p* < 0.0001.

Given that functional crosstalk between bone vascular endothelial cells and the bone‐forming compartment is essential for skeletal homeostasis, we next evaluated angiogenic–osteogenic coupling. Immunofluorescence staining showed that RUNX2^+^ osteoprogenitor cells adjacent to CD31^+^ BVECs were reduced by approximately 50% in OVX‐induced osteoporotic mice. Strikingly, treatment with a TRAIL‐neutralizing antibody restored the osteogenic niche, increasing the RUNX2^+^ cell population by approximately 1.5‐fold compared with control IgG‐treated OVX mice (Figure 6D,E).

We further assessed the effect of TRAIL blockade on osteoclastogenesis, the primary driver of osteoporotic bone resorption. TRAP staining revealed hyperactive osteoclast formation in OVX mice, with osteoclast surface per bone surface increased by approximately 2.4‐fold and osteoclast number per bone surface increased by approximately 2.8‐fold. TRAIL neutralization markedly suppressed this pathological resorption, reducing osteoclast surface and osteoclast number by approximately 41% and 50%, respectively (Figure 6F,G). High‐resolution immunofluorescence staining for ATP6V1B1/B2 further supported this effect. The ATP6V1B1/B2^+^ osteoclast‐associated area adjacent to CD31+ endothelium exhibited a striking 3.0‐fold elevation in OVX mice and decreased by approximately 33% following TRAIL neutralization (Figure 6H,I).

Collectively, these data establish that TRAIL blockade protects against osteoporosis‐associated bone loss by preserving angiogenic–osteogenic coupling and suppressing excessive osteoclastogenesis. These findings further support TRAIL as a functional mediator of endothelial redox dysfunction in osteoporotic bone remodeling.

### 2.7 Endothelial TRX2 Restoration Preserves Type H Vessels and Rescues Osteoporotic Bone Loss

To evaluate whether therapeutic restoration of TRX2 suppresses pathological endothelial TRAIL signaling in vivo, we subjected 12‐week‐old female mice to either OVX or sham surgery. One week after surgery, mice received bilateral intramedullary injection into the femoral bone marrow cavity of AAV particles encoding *Trx2* under an endothelial‐specific promoter (AAV‐TRX2) or a control vector. Femurs were collected for immunofluorescence analysis to assess TRX2 expression and pathway activity. AAV‐TRX2 delivery restored TRX2 expression within the skeletal microenvironment and markedly suppressed TRAIL upregulation in CD31^+^ BVECs of OVX mice (Figure S7A). These data confirm efficient local TRX2 restoration and demonstrate that endothelial TRX2 re‐expression is sufficient to suppress TRAIL activation in osteoporotic bone.

We next evaluated the therapeutic efficacy of TRX2 restoration on bone architecture. Micro‐CT reconstructions revealed that OVX induced pronounced trabecular and cortical bone loss, whereas AAV‐mediated TRX2 delivery substantially reversed these changes (Figure 7A). Quantitative morphometric analysis revealed an approximately 2.0‐fold elevation in bone volume fraction and an approximately 2.1‐fold increase in trabecular number in AAV‐TRX2‐treated OVX mice compared with control vector‐treated OVX mice (Figure 7B). Furthermore, cortical thickness and cortical bone mineral density were also improved after TRX2 restoration (Figure 7C), consistent with preservation of trabecular microarchitecture observed by histological assessments (Figure 7D).

**FIGURE 7:**
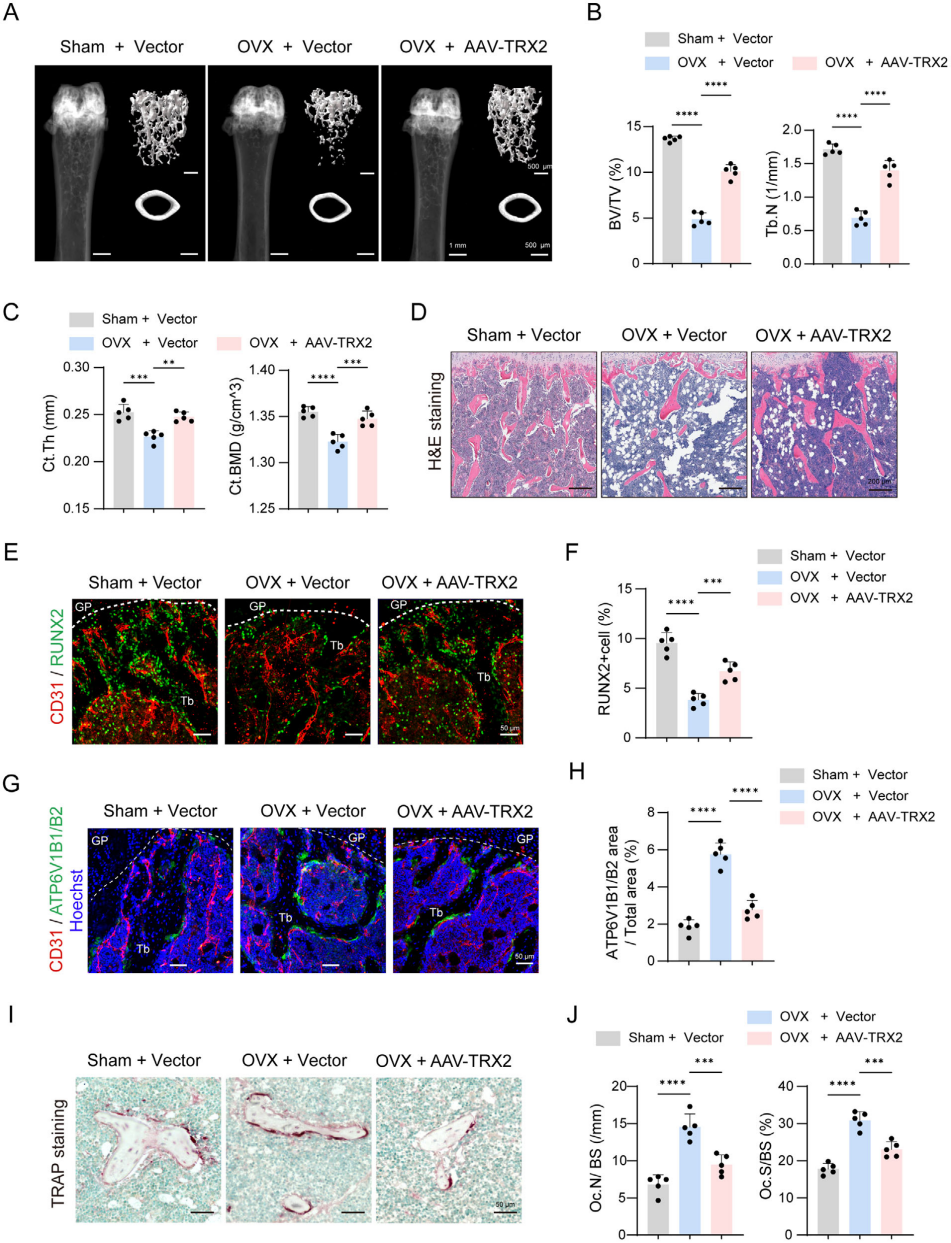
Restoration of endothelial TRX2 preserves osteogenic vessels and attenuates osteoporotic bone loss. A) Representative micro‐CT images of distal femurs from mice in the Sham + Vector, ovariectomy (OVX) + Vector, and OVX + AAV‐TRX2 groups (n = 5). Images include 2D radiographic views of whole femurs (left), three‐dimensional reconstructions of trabecular bone (middle), and cross‐sectional images of cortical bone (right). Scale bars: 1 mm for whole‐femur images and 500 µm for trabecular and cortical bone images. B, C) Quantitative micro‐CT analysis of bone morphometric parameters, including bone volume/tissue volume (BV/TV) and trabecular number (Tb.N) (B), as well as cortical thickness (Ct.Th) and cortical bone mineral density (Ct.BMD) (C). D) Representative H&E–stained femoral sections showing trabecular bone architecture. Scale bars, 200 µm. E, F) Representative immunofluorescence images of CD31^+^ vessels (red) and RUNX2^+^ osteoblast‐lineage cells (green) in the femoral metaphysis (E), with corresponding quantification of the percentage of RUNX2^+^ cells (F). GP, growth plate; Tb, trabecular bone. Scale bars, 50 µm. G, H) Representative immunofluorescence images of CD31^+^ vessels (red), ATP6V1B1/B2^+^ mature osteoclasts (green), and Hoechst‐stained nuclei (blue) in distal femoral sections (G), with corresponding quantification of the ATP6V1B1/B2^+^ area relative to the total tissue area (H). Scale bars, 50 µm. I, J) Representative TRAP–stained distal femoral sections (I) and histomorphometric quantification of osteoclast number per bone surface (Oc.N/BS) and osteoclast surface per bone surface (Oc.S/BS) (J). Scale bars, 50 µm. All quantitative data are presented as means ± SEM. Statistical significance was determined by one‐way analysis of variance (ANOVA) with Tukey's multiple‐comparisons test. ^**^ *p* < 0.01, ^***^ *p* < 0.001, ^****^ *p* < 0.0001.

Within the cellular microenvironment, immunofluorescence staining showed an approximately 1.8‐fold expansion of RUNX2^+^ osteoprogenitors in AAV‐TRX2 ‐treated OVX mice compared with OVX controls (Figure 7E,F). Concurrently, TRX2 restoration reduced osteoclast‐associated ATP6V1B1/B2^+^ area by approximately 50% (Figure 7G,H). TRAP staining further confirmed attenuation of osteoclastogenesis, with approximately 32% and 30% reductions in osteoclast number per bone surface and osteoclast surface per bone surface, respectively (Figure 7I,J). These data establish that TRX2 restoration simultaneously enhances osteogenic activity and suppresses osteoclast‐mediated bone resorption.

Because type H vessels (CD31^hi^EMCN^hi^) are specialized endothelial structures that support osteogenesis [32, 40], we further examined whether TRX2 restoration preserves this vascular niche. Flow cytometric analysis revealed that OVX induced a profound loss of CD31^hi^EMCN^hi^ endothelial cells, with an approximately 90% reduction relative to sham controls. AAV‐mediated TRX2 delivery substantially mitigated this depletion, achieving an approximately 4.5‐fold recovery of the CD31^hi^EMCN^hi^ endothelial population (Figure S7B‐D). In addition, to assess whether endothelial TRX2 regulates the osteogenesis‐supporting program of type H endothelial cells, we isolated CD31^hi^EMCN^hi^ endothelial cells and analyzed their expression of pro‐osteogenic factors. Endothelial *Trx2* deficiency significantly reduced *Bmp4* and *Bmp2* expression, whereas AAV‐mediated TRX2 delivery partially restored both transcripts (Figure S8A‐C), suggesting that TRX2 maintains pro‐osteogenic angiocrine factor expression in type H endothelial cells.

Thus, maintaining the structural and functional homeostasis of these vessels represents a vital therapeutic strategy, and the bone‐protective properties of TRX2 are, at least in part, attributable to its profound capacity to support and sustain this specialized bone‐specific vasculature system.

We also evaluated thein vivo biosafety of the AAV‐TRX2 strategy. Histological evaluation confirmed that the tissue integrity and structural architecture of major visceral organs, including the heart, liver, spleen, lung, and kidney, were completely preserved without any pathological alterations or inflammatory infiltrates (Figure S7E), suggesting good tolerability under the tested conditions.

Taken together, these data demonstrate that endothelial TRX2 restoration attenuates OVX‐induced osteoporotic bone loss by suppressing endothelial TRAIL signaling, restoring RUNX2‐positive osteogenic activity, limiting osteoclast‐mediated bone resorption, and preserving type H vessels. These data support TRX2 as a potential therapeutic target and TRX2‐restoration strategy as a promising intervention for osteoporosis.

## 3 Discussion

The bone vascular niche is increasingly recognized as a central regulator of skeletal homeostasis, yet the mechanisms that connect endothelial dysfunction to osteoporotic bone loss remain incompletely defined [1, 32, 41]. Here, we identify endothelial TRX2 as a mitochondrial redox regulator that preserves skeletal vascular homeostasis, demonstrating that its age‐associated decline in BVECs and endothelial‐specific deletion disrupt osteogenic activity, enhance osteoclastogenesis, and drive trabecular bone loss. Mechanistically, TRX2 directly binds to FOXO3a through a redox‐sensitive Cys90–Cys31 disulfide interaction, anchoring FOXO3a on the mitochondrial outer membrane and preventing its nuclear translocation. Loss of TRX2 or oxidative stress disrupts this interaction, enabling FOXO3a nuclear accumulation and transcriptional activation of TRAIL. TRAIL, in turn, promotes osteoclast differentiation and bone resorption (Figure 8). Importantly, TRAIL neutralization or endothelial TRX2 restoration attenuates bone loss in ovariectomized osteoporotic mice, preserves type H vessels, and rebalances osteogenic and osteoclastogenic activities. Consequently, we define an endothelial TRX2–FOXO3a–TRAIL axis that links mitochondrial redox dysfunction in the skeletal vasculature to osteoporotic bone remodeling imbalance.

**FIGURE 8:**
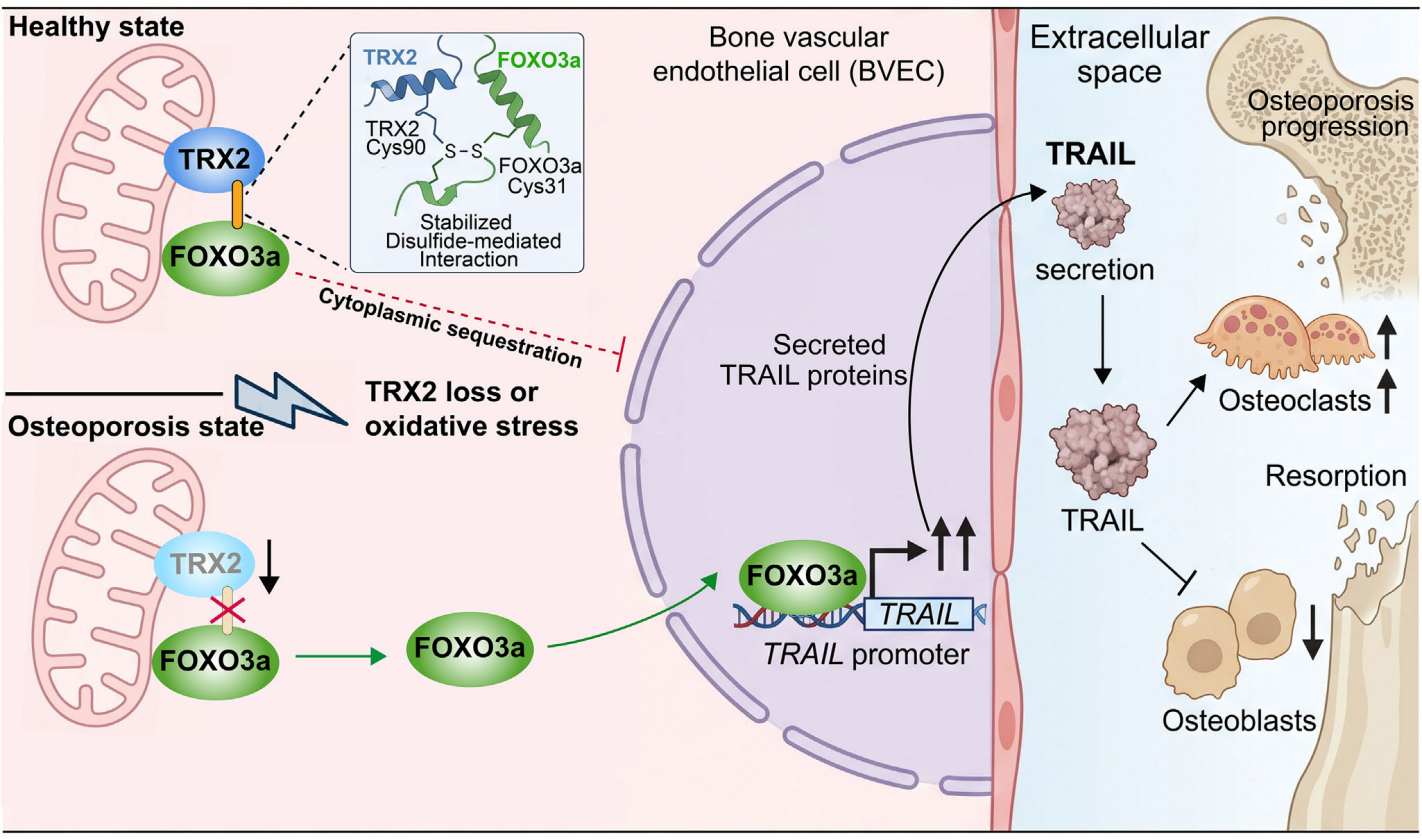
Mechanistic illustration of endothelial TRX2–FOXO3a–mediated TRAIL secretion and osteoclastogenesis in osteoporotic bone loss. In the healthy skeletal vascular niche, TRX2 binds FOXO3a through a redox‐sensitive Cys90–Cys31 disulfide interface, retaining FOXO3a at the mitochondrial outer membrane and preventing its nuclear translocation in BVECs. By contrast, in the osteoporotic state, TRX2 loss or oxidative stress disrupts this disulfide‐mediated interaction, allowing FOXO3a nuclear translocation and activation of the *TRAIL* promoter. Increased endothelial TRAIL secretion remodels the extracellular vascular–skeletal niche by promoting osteoclastogenesis, enhancing osteoclastic bone resorption, and suppressing osteoblast‐associated osteogenesis, thereby driving osteoporosis progression.

Our findings extend the concept of angiogenesis–osteogenesis coupling by defining that endothelial mitochondrial redox state actively controls both osteogenic support and osteoclastogenic signaling within the bone microenvironment. BVECs, particularly CD31hiEMCNhi type H vessels, are documented instructive regulators of angiogenesis–osteogenesis coupling, and their decline during aging, estrogen deficiency, or chronic inflammation, contributes to impaired osteogenesis and osteoporotic bone loss [11, 32, 40, 41, 42]. As a highly conserved mitochondrial thioredoxin, TRX2 maintains endothelial redox homeostasis, suppresses mitochondrial stress, supports angiogenesis and protects against vascular pathology as shown in cardiovascular and retinal vascular contexts [28, 29, 30]. Extending these concepts to bone, we first observed the marked downregulation of TRX2 in BVECs during the pathogenesis of senile osteoporosis. Our study indicates that TRX2 loss in BVECs does not simply weaken the osteogenic support normally provided by type H vessels, but actively converts the vascular niche into a pro‐resorptive microenvironment. Mechanistically, endothelial TRX2 deficiency promotes FOXO3a nuclear translocation and induces TRAIL expression, thereby establishing a non‐cell‐autonomous endothelial FOXO3a–TRAIL axis that enhances osteoclastogenesis. This is distinct from previous studies mainly emphasizing cell‐autonomous FOXO functions in osteoblasts, osteoclasts, or mesenchymal progenitors [43, 44]. Thus, these findings demonstrate that targeted TRX2 restoration mitigates osteoporosis via a bifurcated therapeutic mechanism: it sustains the osteogenic vascular niche by preserving endothelial mitochondrial integrity and type H vessel coupling, while concurrently restraining osteoclastogenesis through the attenuation of FOXO3a/TRAIL signaling. This places TRX2 at the center of a broader paradigm in which mitochondrial redox failure in the bone vasculature is not merely a marker of osteoporosis, but a causal driver of angiogenic deterioration, remodeling uncoupling, and pathological bone loss.

A major mechanistic advance of this study is the identification of a non‐canonical scaffolding function of TRX2. TRX2 is classically viewed as a mitochondrial antioxidant enzyme that maintains redox homeostasis and suppresses stress signaling, including ASK1‐dependent apoptotic pathways [45]. Here, we demonstrate that TRX2 also functions as a redox‐sensitive spatial regulator of transcription factor activity. Rather than regulating total FOXO3a abundance, TRX2 restrains FOXO3a nuclear translocation by maintaining its association with mitochondria. Mutational analyses indicate that TRX2 Cys90 and FOXO3a Cys31 are required for stable complex formation, whereas oxidative challenge disrupts this interaction and promotes FOXO3a nuclear accumulation. This mechanism aligns with broader evidence that FOXO transcription factors are highly sensitive to cellular redox states and that cysteine acquisition contributes to FOXO paralog‐specific redox regulation [46, 47]. Therefore, TRX2 functions not only as a mitochondrial antioxidant enzyme but also as a redox‐sensitive spatial gatekeeper that restrains FOXO3a transcriptional activity.

FOXO transcription factors have been implicated in oxidative stress responses, bone metabolism, osteoblast function, and osteoclast regulation [48, 49, 50]. However, most prior studies have focused on cell‐autonomous effects within osteoblasts, osteocytes, or osteoclast lineage cells [35, 51]. Our findings reveal a non‐cell‐autonomous endothelial mechanism in which FOXO3a activation in bone vascular endothelial cells promotes osteoclastogenesis through TRAIL induction. This distinction is important because it suggests that the skeletal consequences of FOXO3a activation depend on cellular context. In BVECs, FOXO3a nuclear translocation following TRX2 loss is not simply an adaptive antioxidant response; it also activates angiocrine inflammatory signaling that enhances osteoclast formation. The increased TRAIL expression observed in aged endothelial cells and osteoporotic human datasets further supports the pathological relevance of this endothelial FOXO3a–TRAIL axis. Although our findings support endothelial FOXO3a as an important downstream mediator, endothelial‐specific FOXO3a deletion in the OVX model and FOXO3a depletion or ΔNLS‐FOXO3a re‐expression in *Trx2* ^ECKO^ mice were not examined. These complementary models would further define the in vivo role of endothelial FOXO3a and the contribution of its nuclear translocation to TRX2 deficiency‐induced osteoporotic bone loss.

TRAIL represents a compelling downstream therapeutic target for osteoporosis because our findings redefine it as a disease‐specific, vascular‐derived osteoclastogenic factor rather than merely an immune/apoptotic ligand. Although TRAIL has context‐dependent functions in bone biology, earlier studies demonstrated that TRAIL can directly induce osteoclast differentiation from monocyte/macrophage precursors [38], whereas other reports suggested that TRAIL may suppress RANK signaling under specific receptor or microenvironmental conditions by inhibiting lipid raft assembly and TRAF6 recruitment [52]. Our data help clarify these apparently divergent observations by identifying endothelial‐derived TRAIL as a pathological angiocrine mediator in the TRX2‐deficient osteoporotic niche. This finding is clinically relevant because the success of denosumab, a monoclonal antibody targeting RANKL, has already validated excessive ligand‐driven osteoclastogenesis as a major and druggable therapeutic axis in osteoporosis [23, 24, 53, 54]. Therefore, by analogy to RANKL blockade, targeting TRAIL may represent a novel vascular niche–directed antiresorptive strategy for osteoporosis, particularly in patients with endothelial redox dysfunction and elevated TRAIL signaling. Future randomized controlled trials, preceded by rigorous safety, dose‐finding, biomarker‐stratification, and fracture‐endpoint studies, are warranted to determine whether TRAIL neutralization can safely and effectively reduce osteoclast‐driven bone loss and improve clinical outcomes in osteoporotic patients.

Several aspects of these findings warrant further investigation. While the OVX model provides a rapid and reproducible platform for evaluating estrogen deficiency‐induced bone loss, studies in naturally aged mice would offer complementary evidence regarding the therapeutic effects of endothelial TRX2 restoration in the progressive and multifactorial context of age‐related osteoporosis. Future work may also clarify whether TRX2 contributes to mesenchymal‐like phenotypic changes in aging BVECs. In addition, lineage‐tracing and ex vivo differentiation approaches may help determine whether TRAIL blockade or endothelial TRX2 restoration directly influences bone marrow mesenchymal stromal cells (BMSC) lineage commitment.

In summary, our study identifies endothelial TRX2 as a critical mitochondrial safeguard of the skeletal vascular niche. By restraining FOXO3a nuclear translocation through a redox‐sensitive Cys90–Cys31 interaction, TRX2 prevents pathological TRAIL production and preserves the coupling between angiogenesis, osteogenesis, and osteoclast regulation. These findings broaden the function of TRX2 from a canonical antioxidant enzyme to a spatial regulator of redox‐sensitive transcriptional signaling and support therapeutic targeting of the endothelial TRX2–FOXO3a–TRAIL axis as a potential strategy for osteoporosis.

## 4 Materials and Methods

### 4.1 scRNA‐seq and Transcriptomic Data Analysis

Publicly available scRNA‐seq data (BioProject: PRJNA1000447) were obtained from the BioProject database [31]. After conversion to FASTQ format using the SRA Toolkit, reads were aligned to the GRCm38 mouse reference genome and quantified using Cell Ranger (v4.0.10, 10x Genomics). Ambient background RNA was removed using CellBender (v0.3.0) [55]. Downstream analyses, including quality control, normalization, dimensionality reduction, clustering, and visualization, were performed using Scanpy (v1.9.6) [56].

Cells were retained if they met the following criteria: more than 500 detected genes, fewer than 70,000 total unique molecular identifier (UMI) counts, and less than 25% mitochondrial gene content. For feature selection, the top 4,000 highly variable genes were selected using the function *highly_variable_genes* with the parameters *flavor = “seurat_v3”* and *batch_key = “sample”*, while excluding cell cycle‐related genes as previously reported [31]. To integrate datasets, single‐cell transcriptomes were projected into a batch‐corrected, low‐dimensional latent space using the scVI model, with each individual sample defined as a separate batch [57]. Doublets were identified and removed using the SOLO implementation in scvi‐tools (v1.0.3) [58]. For visualization, the scVI latent representation was used to construct a neighborhood graph, followed by dimensionality reduction using uniform manifold approximation and projection (UMAP). Unsupervised clustering was performed using the Leiden algorithm, and cell types were annotated according to canonical markers [31, 59]. Non‐endothelial populations lacking *Emcn* and *Pecam1* expression were excluded from downstream analyses.

RNA velocity was estimated using Velocyto (v0.17.15) to quantify spliced and unspliced transcripts, and trajectories were projected onto UMAP embeddings via scVelo (v0.3.2) [60, 61]. Transcription factor regulatory activities and cytokine signaling activities were inferred using decoupler (v1.5.0) with DoRothEA and CytoSig resources, respectively [62, 63, 64]. Differentially expressed genes (DEGs) were identified using thresholds of |log_2_ fold change| > 0.5 and p < 0.0001, followed by Gene Ontology (GO) enrichment analysis using the DAVID web server [65]. For bulk transcriptomic analysis, the microarray dataset GSE230665, which contains iliac bone samples from 12 patients with postmenopausal osteoporosis and 3 healthy controls, was obtained from the Gene Expression Omnibus (GEO) database [66]. DEGs were identified using the GEO2R interactive web tool [67].

### 4.2 Animal Model Establishment and Treatment

C57BL/6 mice were obtained from the Laboratory Animal Center of Guangdong Province. *Trx2* ^fl/fl^ mice were constructed and produced by the Shanghai Model Organisms Center (Shanghai, China). To generate endothelial cell‐specific Trx2 knockout mice, *Trx2* ^fl/fl^ mice were crossed with *Cdh5*‐*Cre* transgenic mice obtained from the Shanghai Model Organisms Center (Shanghai, China). The resulting *Trx2* ^fl/fl^; *Cdh5‐Cre^+^* mice are hereafter referred to as *Trx2* ^ECKO^ mice. Littermate *Trx2* ^fl/fl^ (*Cre*‐negative) mice served as the control group (*Trx2* ^WT^). Genotyping was performed via PCR analysis of genomic DNA extracted from tail biopsies. All animal experiments were approved by the Institutional Animal Care and Use Committee of the Second Affiliated Hospital of South China University of Technology (approval no. K‐2022‐014‐01) and conducted in accordance with institutional guidelines and the National Institutes of Health Guide for the Care and Use of Laboratory Animals.

To establish the postmenopausal osteoporosis model, 12‐week‐old female mice underwent bilateral ovariectomy (OVX) or sham surgery under general anesthesia. For endothelial‐targeted gene therapy, Adeno‐associated virus 9 (AAV9) vectors for encoding *Trx2* under the control of the *Tie2* promoter (AAV‐Tie2‐Trx2) or negative control vectors were constructed and produced by GeneChem (Shanghai, China). One week after OVX, mice received bilateral intramedullary injections of AAV vectors into the femoral bone marrow cavities at a viral titer of 1.12 × 10^13^ vg/mL. Samples were collected 5 weeks after injection.

For pharmacological TRAIL neutralization, OVX mice were treated with a TRAIL‐neutralizing antibody (5 mg/kg; Thermo Fisher Scientific, 16‐5951‐85) or an isotype IgG control antibody (5 mg/kg; Thermo Fisher Scientific, 16‐4321‐82) by tail vein injection every 3 days for 4 weeks, beginning 1 week after surgery. For early postnatal intervention, postnatal day 7 (P7) *Trx2* ^ECKO^ and *Trx2* ^WT^ mice received intraperitoneal injections of the TRAIL‐neutralizing antibody or isotype control every 3 days until P21.

### 4.3 Microcomputed Tomography

Mice were euthanized, and femurs and tibias were dissected and fixed in 4% paraformaldehyde (PFA) overnight at 4°C. The isolated bones were scanned using a Bruker micro–computed tomography (micro‐CT) scanner. Three‐dimensional (3D) reconstructions were generated and visualized using DataViewer (v1.7.0.1, Bruker), and CTvox (v3.3.1, Bruker) software. For trabecular bone analysis, the region of interest (ROI) was defined as the metaphyseal region starting 0.5 mm proximal to the distal growth plate and extending 2.5 mm proximally. For cortical bone analysis, the ROI was defined at the mid‐diaphysis and extended longitudinally for 0.5 mm. Bone morphometric parameters, including bone volume fraction (BV/TV), trabecular thickness (Tb.Th), trabecular number (Tb.N), trabecular separation (Tb.Sp), and cortical thickness (Ct.Th), were quantified using CTAn software (v1.23, Bruker).

### 4.4 Bone Immunofluorescence and Histology

For immunofluorescence (IF), femurs were fixed in 4% paraformaldehyde (PFA) overnight at 4°C, rinsed with PBS, and decalcified in 0.5 M EDTA for 3 days. Tissues were dehydrated in 20% sucrose/2% polyvinylpyrrolidone (PVP; TargetMol), embedded in optimal cutting temperature (OCT) compound (Sakura), and cryosectioned according to the experimental purpose. Specifically, 5 µm sections were used for TRX2 and TRAIL staining, while 50 µm sections were used for CD31/RUNX2 and ATP6V1B1/B2 staining. Sections were treated with an autofluorescence quencher (Servicebio, G1221) and permeabilized with 0.3% Triton X‐100. Following three 10‐min washes with PBS, sections were blocked with 5% donkey serum for 1 h at room temperature. Sections were then incubated overnight at 4°C with primary antibodies diluted in a solution containing 5% donkey serum in PBS. Primary antibodies were: anti‐TRX2 (1:200, Abcam, ab185544), anti‐CD31 (1:100, R&D Systems, AF3628), anti‐RUNX2 (1:200, Cell Signaling Technology, 12556), anti‐ATP6V1B2 (1:200, Abcam, ab200839), anti‐TRAIL (1:100, Thermo Fisher Scientific, N2B2), anti‐Flag (1:200, Sigma–Aldrich, F1804), anti‐FOXO3a (1:200, Proteintech, 10849‐1‐AP). After primary antibody incubation, sections were washed with PBS three times at 10‐min intervals and then incubated for 1 h at room temperature in the dark with suitable secondary antibodies diluted in 5% donkey serum. Secondary antibodies were: anti‐goat Alexa Fluor 594 (1:300, Thermo Fisher Scientific, a‐11058), anti‐rabbit Alexa Fluor 647 (1:400, Abcam, ab150075), anti‐mouse Alexa Fluor 647 (1:400, Abcam, ab150107), anti‐rat Alexa Fluor 647 (1:300, Thermo Fisher Scientific, A48272TR) and anti‐rabbit Alexa Fluor 488 (1:400, Thermo Fisher Scientific, A‐21206). Sections were then washed with PBS and mounted with an antifade mounting medium containing Hoechst (Beyotime, P0133), and imaged using a fluorescence microscope (Zeiss).

For histological analysis, fixed femurs were decalcified in 0.5 M EDTA for 3 to 4 weeks, dehydrated through a graded ethanol series, cleared in xylene, and embedded in paraffin. Sections were cut at 5 µm thickness and stained with hematoxylin and eosin (H&E; Solarbio, G1120) or tartrate‐resistant acid phosphatase (TRAP; Servicebio, G1050) according to the manufacturers’ instructions.

### 4.5 Cell Isolation and Culture

For primary cell isolation, bone marrow cells were harvested from the femurs and tibiae of 6‐week‐old mice. Briefly, bone marrow cells were flushed from the bone marrow cavity and cultured in α‐minimum essential medium (α‐MEM; Gibco) supplemented with 10% fetal bovine serum (FBS), 1% penicillin‐streptomycin, and macrophage colony‐stimulating factor (M‐CSF; 30 ng/ml, Sino Biological). After 3 days of culture, adherent cells were collected as bone marrow‐derived macrophages (BMMs) for subsequent experiments.

Human umbilical vein endothelial cells (HUVECs), HEK293T cells, and C166 mouse endothelial cells were obtained from the American Type Culture Collection (ATCC). HEK293T and C166 cells were maintained in Dulbecco's modified Eagle's medium (DMEM; Gibco) supplemented with 10% FBS and 1% penicillin‐streptomycin. HUVECs were cultured in endothelial cell medium (ScienCell) supplemented with the endothelial cell growth supplement. All cells were maintained at 37°C in a humidified incubator with 5% CO_2._

### 4.6 Plasmids and Constructs

DNA sequences encoding the wild‐type genes were purchased from GenScript, amplified by polymerase chain reaction (PCR), and inserted into different vectors. All PCR‐amplified fragments and linearized vector backbones with homologous ends were seamlessly assembled using the ClonExpress II One Step Cloning Kit (Vazyme, C112‐01) according to the manufacturer's instructions. All constructed plasmids and mutational variants are listed in Table S1, and their accuracy was validated by Sanger sequencing.

### 4.7 Lentiviral Package and Cell Transduction

To produce lentiviruses, HEK293T cells seeded in 10‐cm dishes were co‐transfected with 10 µg of the lentiviral transfer vector expressing the target gene/shRNA, 10 µg of psPAX2, and 4 µg of pMD2.G using PEI transfection system (Polysciences, 24765‐1). 48 h after transfection, the culture supernatant containing the lentivirus was harvested and filtered through a 0.45‐µm pore‐size filter, and concentrated using polyethylene glycol 6000 (PEG6000; Aladdin). Cells were then transduced with the lentivirus supplemented with 8 µg/mL polybrene (Beyotime) for 6–8 h. At 48 h post‐transduction, the cells were harvested for subsequent experiments. The transduction efficiency was assessed by flow cytometry and target gene expression was verified via western blot. The lentiviral transfer vectors constructed in this study are detailed in Table S1.

### 4.8 RNA Interference

For RNA interference, cells were transfected with the indicated small interfering RNAs or a non‐targeting control siRNA using RNAiMAX (Thermo Fisher Scientific, 13778150) according to the manufacturer's instructions. The siRNA sequences used in this study are listed in Table S2.

### 4.9 Cell Fluorescence Imaging and Co‐Localization Analysis

To determine the subcellular localization and co‐localization of TRX2 and FOXO3a, HEK293T cells were seeded in tissue culture–treated glass‐bottom dishes (Cellvis, D35‐20‐1.5P). When cells reached 50% to 60% confluence, expression vectors encoding RFP‐tagged TRX2 and GFP‐tagged FOXO3a (1 µg each) were co‐transfected using GoldenTran‐D reagent (Changchun Golden Transfer Science and Technology Co., Ltd.; 160326015) according to the manufacturer's instructions. Twenty‐four hours after transfection, cells were incubated with MitoTracker Deep Red (Thermo Fisher Scientific, A66440) and Hoechst 33342 (Beyotime, C1022) at 37°C for 30 min to label mitochondria and nuclei, respectively. Cells were then fixed with 4% paraformaldehyde for 15 min.

To assess the effect of TRX2 on FOXO3a localization, cells were transfected with TRX2 siRNA (siTRX2; target sequence: 5′‐GGATGGACCTGACTTTCAA‐3′) or a non‐targeting control siRNA (siCon) at a final concentration of 100 nm using Lipofectamine RNAiMAX (Thermo Fisher Scientific, 13778150) for 48 h. After mitochondrial labeling with MitoTracker Deep Red, endogenous FOXO3a was detected by immunofluorescence staining with an anti‐FOXO3a primary antibody (1:300, Proteintech, 10849‐1‐AP) and an Alexa Fluor 488–conjugated secondary antibody. Nuclei were counterstained with Hoechst 33342. Fluorescence images were acquired using structured illumination microscopy (SIM; Zeiss). Spatial colocalization was analyzed using the Coloc 2 plugin in ImageJ software (v1.54p).

### 4.10 In Vitro Osteoclastogenesis and Co‐Culture

For the monoculture osteoclastogenesis assay, BMMs were isolated from *Trx2* ^ECK^ *^O^* and *Trx2* ^WT^. Cells were seeded in 24‐well plates at a density of 3 × 10^4^ cells/well and stimulated with M‐CSF (30 ng/mL, Sino Biological) and RANKL (50 ng/mL, R&D).

For the coculture system, BMMs were seeded in the lower chamber of a Transwell plate and stimulated with M‐CSF and RANKL to induce osteoclast differentiation. Endothelial cells transfected or stably transduced with the indicated constructs were seeded in the upper chamber. Where indicated, a TRAIL neutralizing antibody (5 µg/mL) or an IgG isotype control was added to the media. Cells were subjected to TRAP staining 5–7 days after induction.

### 4.11 Subcellular Fractionation and Protein Extraction

For the analysis of the regulatory effect of TRX2 on the subcellular distribution of FOXO3a, cells were transfected with either siTRX2 or siCon at final concentrations of 0, 25, or 100 nm, as indicated, for 48 h. Subsequently, cytoplasmic and nuclear fractions were isolated using a nuclear extraction kit (PC204, Epizyme Biotech) according to the manufacturer's instructions. The isolated protein fractions were immediately subjected to western blot analysis.

### 4.12 Immunoblots

Whole‐cell lysates were extracted using lysis buffer supplemented with protease inhibitors. Protein concentrations were determined using a BCA assay (Thermo Fisher Scientific, #23225). Equal amounts of proteins were separated by SDS‐PAGE and transferred onto nitrocellulose membranes using the Trans‐Blot Turbo Transfer System (Bio‐Rad). Membranes were blocked with 5% non‐fat milk for 1 h at room temperature and subsequently incubated overnight at 4°C with primary antibodies. Primary antibodies included: TRX2 (1:5000, Invitrogen, PA5‐81102), TRX2 (1:5000, Abcam, ab185544), TRAIL (1:2000, Proteintech, 66756‐1‐Ig), TRAIL (1:2000, Thermo Fisher Scientific, 1J5S5), FOXO3a (1:3000, Proteintech, 10849‐1‐AP), VDAC1 (1:5000, Proteintech, 10866‐1‐AP), HSP60 (1:5000, Proteintech,15282‐1‐AP), Histone H3 (1:5000, Proteintech, 68345‐1‐Ig), Lamin B1 (1:5000, Selleck, F0523), Flag (1:5000, Beyotime, AF2855), HA (1:5000, Proteintech, HRP‐66006), GAPDH (1:20000, Servicebio, ZB15004), and β‐Actin (1:20000, Servicebio, ZB15001). Blots were then incubated with HRP‐conjugated secondary antibodies and visualized using an ECL substrate.

### 4.13 Co‐Immunoprecipitation (Co‐IP)

To investigate the interaction between TRX2 and FOXO3a, we constructed different tag‐conjugated constructs, including HA‐tagged TRX2, Flag‐tagged TRX2 and Flag‐tagged FOXO3a. To identify the specific binding sites or domains responsible for this interaction, we further constructed different cysteine‐to‐serine mutational variants, including HA‐tagged TRX2‐C90S, TRX2‐C93S, and TRX2‐C90/93S, as well as Flag‐tagged FOXO3a‐C31S, FOXO3a‐ΔCys, and FOXO3a‐ΔCys‐C31. Here, FOXO3a‐ΔCys represents a fully substituted variant where all cysteine residues within the target region were replaced with serines, while FOXO3a‐ΔCys‐C31 serves as a single‐cysteine retention mutant that incorporates the same all‐serine background but leaves only the cysteine at position 31 intact.

Approximately 48 h post‐transfection, cells within the 6‐cm dishes were harvested and lysed in ice‐cold Pierce IP Lysis Buffer (Thermo Fisher Scientific) supplemented with a protease inhibitor cocktail. Cell lysates were immunoprecipitated with anti‐Flag M2 affinity beads (Sigma‐Aldrich) and incubated overnight at 4°C with constant rotation. After washing with STN buffer (10 mM Tris‐HCL, 0.5% NP‐40, 150 mM NaCl, 0.5% TritonX‐100, ddH_2_O) to remove non‐specific binding, the bound immune complexes were eluted by boiling the beads in SDS‐PAGE loading buffer at 95°C for 10 min. For mass spectrometry (MS) analysis, the beads were further rinsed three times with cold PBS to remove residual detergents and then submitted to Shanghai Applied Protein Technology Co., Ltd. for further experiments and analyses. The mass spectrometry data generated in this study have been deposited in Zenodo at https://doi.org/10.5281/zenodo.22741153.

### 4.14 Proximity Ligation Assay (PLA)

Cells were seeded in glass‐bottom confocal dishes and transfected the following day with 100 nM siTRX2 or siCon. After 48 h, cells were fixed with 4% paraformaldehyde for 15 min and permeabilized with 0.1% Triton X‐100. Protein–protein interactions were then detected by in situ proximity ligation assay using the Duolink In Situ Orange Starter Kit Mouse/Rabbit (Sigma‐Aldrich, DUO92102) according to the manufacturer's instructions. After blocking, cells were incubated overnight at 4°C with primary antibodies anti‐TRX2 (1:200, Abcam, ab185544) and anti‐FOXO3a (1:200, Proteintech, 66428‐1‐Ig), followed by anti‐rabbit PLUS and anti‐mouse MINUS PLA probes, ligation, and amplification. Nuclei were counterstained with Hoechst, and PLA signals were visualized by confocal fluorescence microscopy (Zeiss).

### 4.15 Proteinase K Protection Assay

Mitochondria were isolated using a Mitochondrial Isolation Kit (Epizyme, PC205). The isolated mitochondria were incubated with proteinase K (PK; 10 µg/mL; Vazyme) on ice for 30 min in the presence or absence of 1% Triton X‐100. The reaction was subsequently terminated by the addition of a protease inhibitor cocktail, followed by western blot analysis.

### 4.16 Enzyme‐Linked Immunosorbent Assay (ELISA)

Cell culture supernatants were collected and centrifuged. Samples diluted in CBS buffer were coated onto 96‐well plates overnight at 4°C. Plates were washed with PBST, blocked with 1% BSA for 1 h, and incubated sequentially with anti‐TRAIL (1:1000, Proteintech, 66756‐1‐Ig), biotin‐conjugated secondary antibody (1:10000, Proteintech, SA00004‐1), and HRP‐conjugated streptavidin (1:5000, Proteintech, SA00001‐0). After addition of TMB substrate (Beyotime, P0209), the reaction was stopped, and absorbance was measured at 450 nm using a microplate reader (Thermo Fisher Scientific).

### 4.17 Protein‐Protein Interaction Prediction

Human TRX2 and FOXO3a protein sequences were retrieved from the UniProt database. The structure of the TRX2–FOXO3a complex was predicted using AlphaFold3 with default parameters [68]. The DOCKQ score was calculated to evaluate the quality of the protein‐protein interface according to previously established metrics [69]. The resulting 3D models were visualized and analyzed using PyMOL (v3.1.0).

### 4.18 Flow Cytometry and Cell Sorting

Fresh femora and tibiae were crushed in ice‐cold PBS and digested with 2.5 mg/mL Collagenase A (Roche) and 1 U/mL Dispase II (Roche) for 20 min at 37°C. Digested cells were filtered through a 70‐µm strainer and treated with RBC lysis buffer (Solarbio) to obtain single‐cell suspensions. Equal numbers of cells were stained with BV510‐CD45 (Biolegend, 103138), PE‐Cy7‐Ter119 (Invitrogen, 25‐5921‐81), PE‐CD31 (Invitrogen, 12‐0311‐81), and AF647‐Endomucin (Santa Cruz, sc‐65495) antibodies for 45 min at 4°C in the dark. 7‐AAD was added 5 min before acquisition to exclude dead cells. Flow cytometric analysis and cell sorting were performed on BD LSRFortessa and FACSAria III cytometers, respectively. Data were analyzed using FlowJo software (v10.4).

### 4.19 RNA Extraction and RT‐qPCR

Total RNA was extracted from ECs using the EZ‐press RNA Purification Kit (EZBioscience, B0004D) according to the manufacturer's instructions. RNA was reverse‐transcribed into cDNA using the Reverse Transcription Kit (EZBioscience, A0010CGQ). Quantitative PCR (qPCR) was performed using qPCR SYBR Green Master Mix (EZBioscience, A0012‐R2) on a QuantStudio 5 Real‐Time PCR System (Thermo Fisher Scientific). Primer sequences used for qPCR are listed in Table S2.

### 4.20 CUT&Tag and Sequencing

CUT&Tag was performed using the Hyperactive Universal CUT&Tag Assay Kit (Vazyme) according to the manufacturer's instructions. Briefly, freshly sorted ECs (2 × 10^4^ cells per reaction) were bound to concanavalin A–coated magnetic beads and incubated overnight at 4°C with a rabbit anti‐FOXO3a monoclonal antibody (1:50; Cell Signaling Technology, #2497), followed by incubation with a goat anti‐rabbit secondary antibody. After binding of the pA/G–Tn5 transposome, tagmentation was performed at 37°C for 1 h, and the tagmented DNA was purified and PCR‐amplified to generate sequencing libraries. Libraries were sequenced on an Illumina HiSeq 2500 platform with 150‐bp paired‐end reads.

Sequencing reads were trimmed using Trim Galore (v0.6.6) and aligned to the mouse GRCm38 reference genome using Bowtie2 (v2.4.4) with default parameters. PCR duplicates were removed using Picard (v3.1.0), followed by removal of low‐quality and secondary alignments. The resulting BAM files were coordinate‐sorted and indexed using SAMtools (v1.19.2). To enable direct comparisons across samples, spike‐in reads were used as an internal reference to calculate sample‐specific scaling factors for signal normalization.

For validation of FOXO3a occupancy, tagged DNA obtained from CUT&Tag assays was subjected to qPCR using primers targeting the indicated genomic regions. Primer sequences used for CUT&Tag‐qPCR are listed in Table S2.

### 4.21 Statistical Analysis

Immunofluorescence, immunoblot, H&E staining, TRAP staining and micro‐CT analysis quantifications were performed using ImageJ (v1.54p) and CTAn (v1.23, Bruker) software. All analyses were performed using the GraphPad Prism 9 software (GraphPad Software Inc., San Diego, CA, USA). Group comparisons were made using a two‐tailed unpaired Student's t‐test, one‐way or two‐way ANOVA followed by Tukey's test. All the error bars represent the standard error of the mean (SEM). P‐values < 0.05 were considered significant: ^*^ *p* < 0.05; ^**^ *p* < 0.01; ^***^ *p* < 0.001; ^****^ *p* < 0.0001. ns, no significant difference.

## Author Contributions

C.Z., Z.Z., and Y.D. contributed equally to this work. F.H., Z.S., and Z.L. performed conception of the hypothesis. F.H., Z.S., Z.L., and Z.T. performed study supervision. C.Z., Z.Z., Y.D., and X.L. performed development of methodology. C.Z., Z.Z., Y.D., X.L., R.L., and X.Z. performed the acquisition of data. C.Z., Z.Z., Y.D., X.L., R.L., X.Z. J.C., and Z.T. performed analysis and interpretation of data. C.Z., Y.D., F.H., Z.T., Z.S., and Z.L. wrote, reviewed, and/or revised the manuscript. All authors discuss the study. All authors have given approval to the final version of the manuscript.

## Funding

This study was supported by grants from the National Natural Science Foundation of China (Nos. 82470748, 82070752, 82170939), Guangdong Basic and Applied Basic Research Foundation (Nos. 2022B1515020106, 2023A1515012477), and the Guangdong Provincial Department of Science and Technology (No. 202201020273).

## Conflicts of Interest

The authors declare no conflicts of interest.

## Supporting information




**Supporting File**: advs78304‐sup‐0001‐SuppMat.pdf.

## Data Availability

The mass spectrometry data generated in this study have been deposited in the Zenodo repository with the DOI 10.5281/zenodo.22741153. All other data are available from the corresponding authors upon request.
