# Supplementary material for: Thioredoxin‐2 Alleviates Osteoporosis by Restraining FOXO3a Nuclear Translocation
Source: Adv Sci (Weinh). 2026 Oct 18:e78304. Online ahead of print. doi: 10.1002/advs.78304 (PMC13676988; doi:10.1002/advs.78304)
Supplement: Supplementary file 1 [file ADVS-9999-e78304-s001.pdf]

Supporting Information

Thioredoxin-2 Alleviates Osteoporosis by Restraining FOXO3a Nuclear Translocation

Chi Zhan[1, 2], Zhaocai Zhou[2], Yue Ding[1], Rong Lian[1], Xinyu Li[1], Xintong Zhang[2], Jie Cao[1], Zhonghui Tang[3], Zhengmei Lin[2]*, Zhi Song[2]*, Feng He[1]*

This PDF file includes:

Figures S1 to S10

Tables S1 to S2

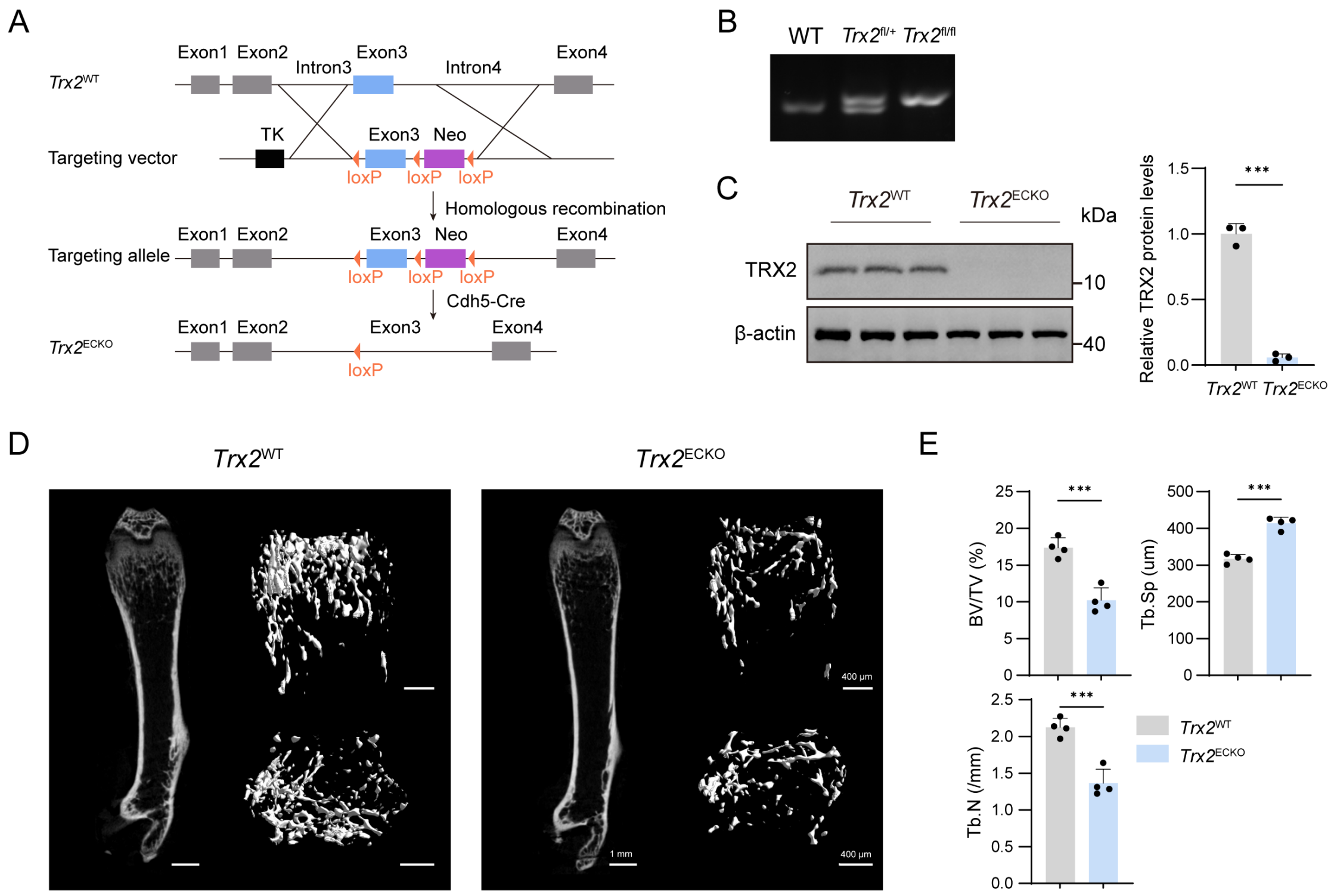

Figure S1. Generation and characterization of endothelial cell–specific Trx2 knockout mice. A) Schematic illustration of the strategy used to generate endothelial cell–specific Trx2 knockout (*Trx2*^{ECKO}) mice using the Cre-loxP recombination system. Exon 3 of the *Trx2* gene was flanked by loxP sites and deleted by crossing *Trx2*$^{fl/fl}$ mice with Cdh5-Cre transgenic mice. B) Representative polymerase chain reaction (PCR) genotyping results using tail genomic DNA from wild-type (WT), heterozygous (*Trx2*$^{fl/+}$), and homozygous floxed (*Trx2*$^{fl/fl}$) mice. C) Representative Western blot images and quantification of TRX2 protein levels in 3-week-old *Trx2*^{WT} and *Trx2*^{ECKO} mice. β-actin served as the loading control (n = 3). D) Representative micro–computed tomography (micro-CT) images of femurs from 6-week-old *Trx2*^{WT} and *Trx2*^{ECKO} mice. Images show two-dimensional longitudinal sections (left) and three-dimensional reconstructions of trabecular bone (right) (n = 4). Scale bars, 1 mm for longitudinal images and 400 μm for three-dimensional reconstructions. E) Quantitative micro-CT analysis of trabecular bone volume/tissue volume (BV/TV), trabecular separation (Tb.Sp), and trabecular number (Tb.N) in femurs from 6-week-old mice. Source data for the unedited raw immunoblots

shown in Figure S10. All quantitative data are presented as means ± SEM. Statistical significance was determined by two-tailed unpaired Student's t test. ***$P < 0.001$.

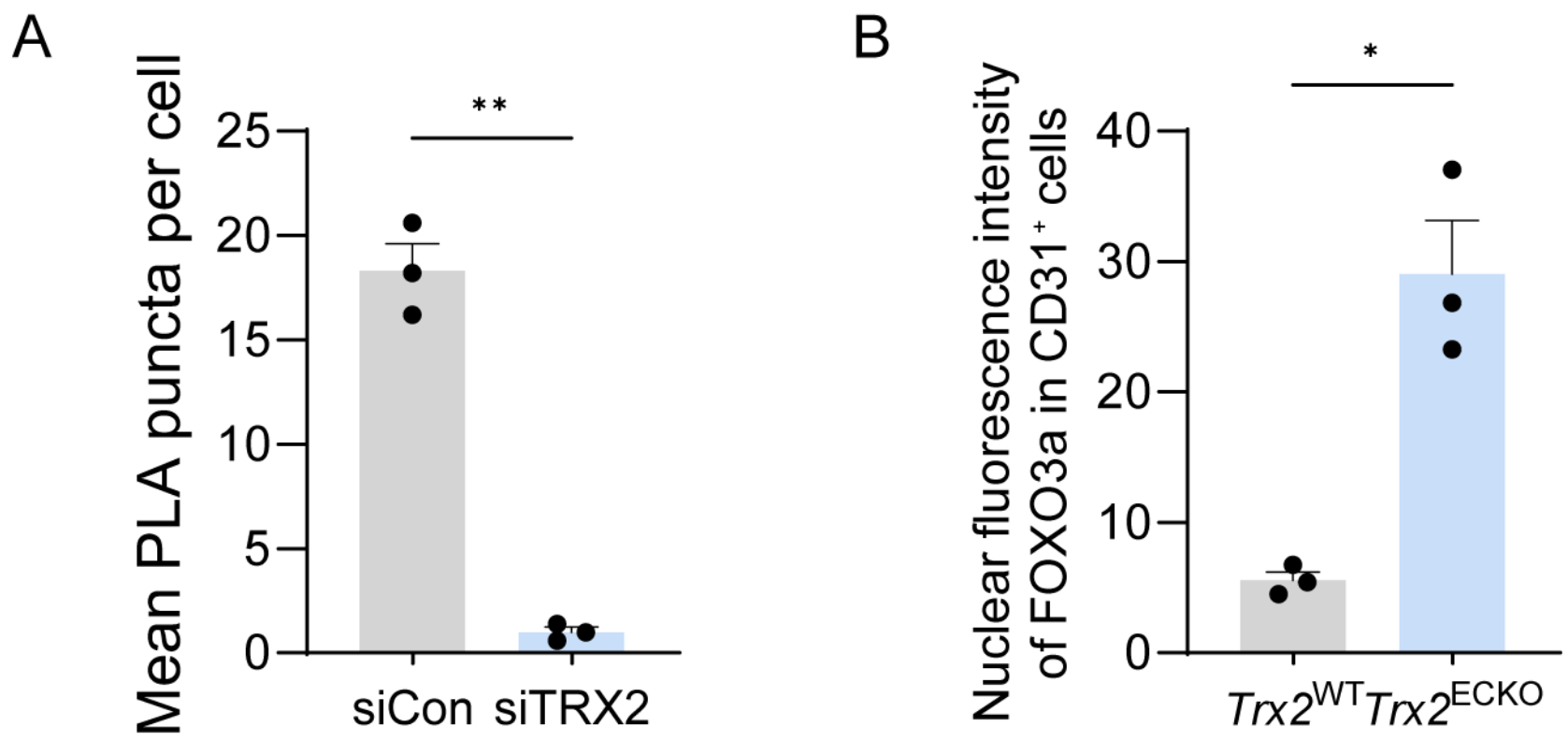

Figure S2. Endothelial TRX2 deficiency disrupts TRX2-FOXO3a proximity and promotes FOXO3a nuclear accumulation. A) Quantification of TRX2–FOXO3a proximity signals shown in Figure 4D, expressed as the number of Duolink proximity ligation assay (PLA) puncta per cell in siCon- and siTRX2-treated HUVECs. B) Quantification of nuclear FOXO3a fluorescence intensity in $CD31^+$ BVECs corresponding to Figure 4E. All quantitative data are presented as means ± SEM. Statistical significance was determined by two-tailed unpaired Student's t test. *$P < 0.05$, **$P < 0.01$.

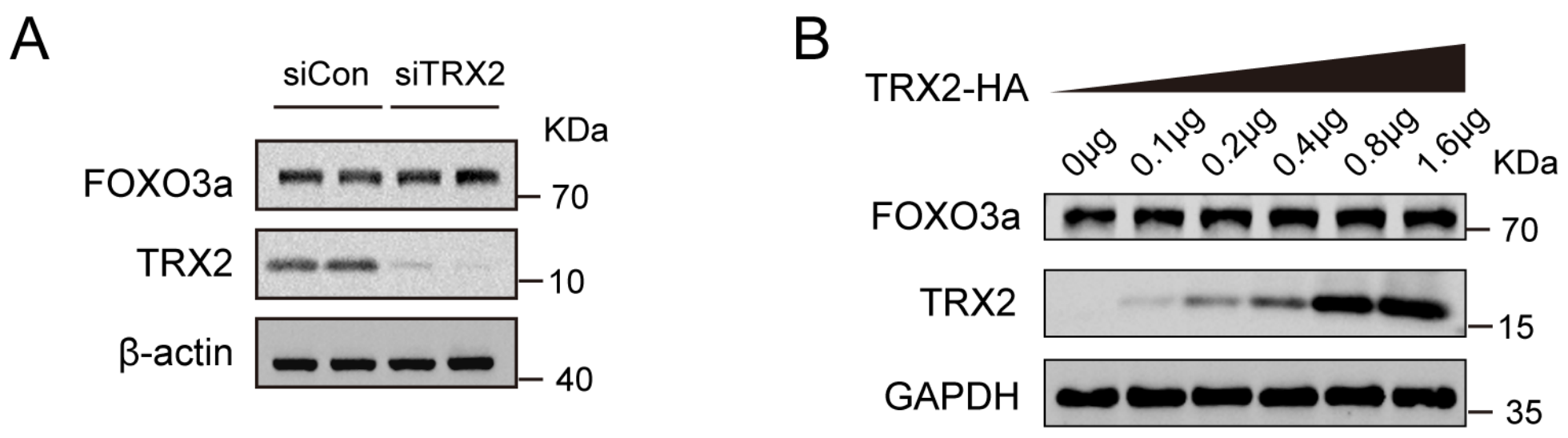

Figure S3. TRX2 does not affect total FOXO3a protein abundance. A) Western blot analysis of FOXO3a and TRX2 protein levels in HEK293T cells transfected with negative control siRNA (siCon) or TRX2-specific siRNA (siTRX2). β-actin served as the loading control. B) Western blot analysis of FOXO3a and TRX2 protein levels in HEK293T cells transfected with increasing amounts of the TRX2-HA overexpression plasmid: 0, 0.1, 0.2, 0.4, 0.8, and 1.6 μg. GAPDH served as the loading control. Source data for the unedited raw immunoblots shown in Figure S10.

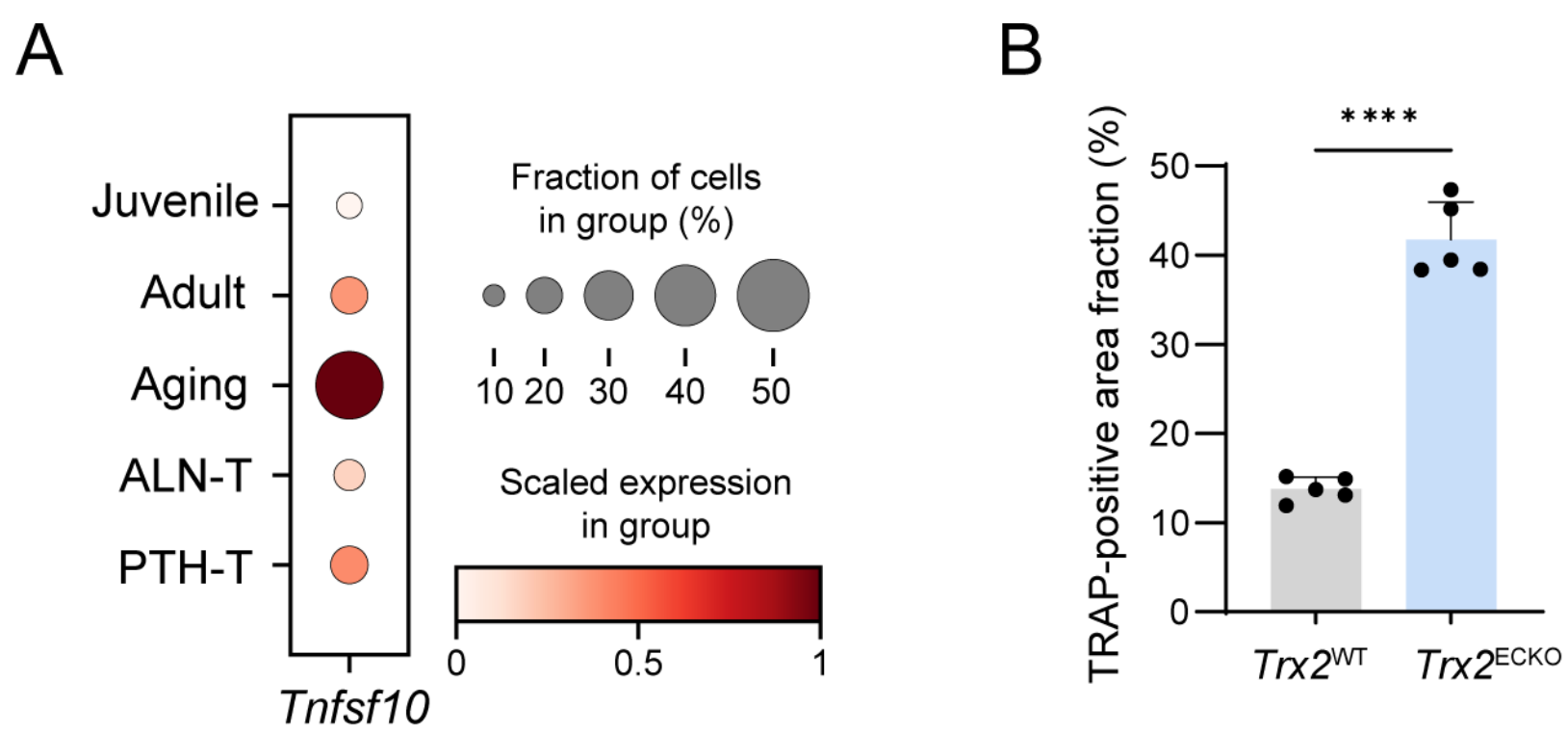

Figure S4. Single-cell RNA sequencing analysis reveals increased *Tnfsf10* expression in aging bone vascular endothelial cells. A) Dot plot showing the expression pattern of *Tnfsf10* in bone vascular endothelial cells across the indicated groups, including Juvenile, Adult, Aging, ALN-T, and PTH-T. Dot size indicates the fraction of cells expressing *Tnfsf10* within each group, and color intensity indicates scaled average expression. B) Quantification of the tartrate-

resistant acid phosphatase (TRAP)–positive area fraction corresponding to Figure 5F. All quantitative data are presented as means ± SEM. Statistical significance was determined by two-tailed unpaired Student's t test. ****P < 0.0001.

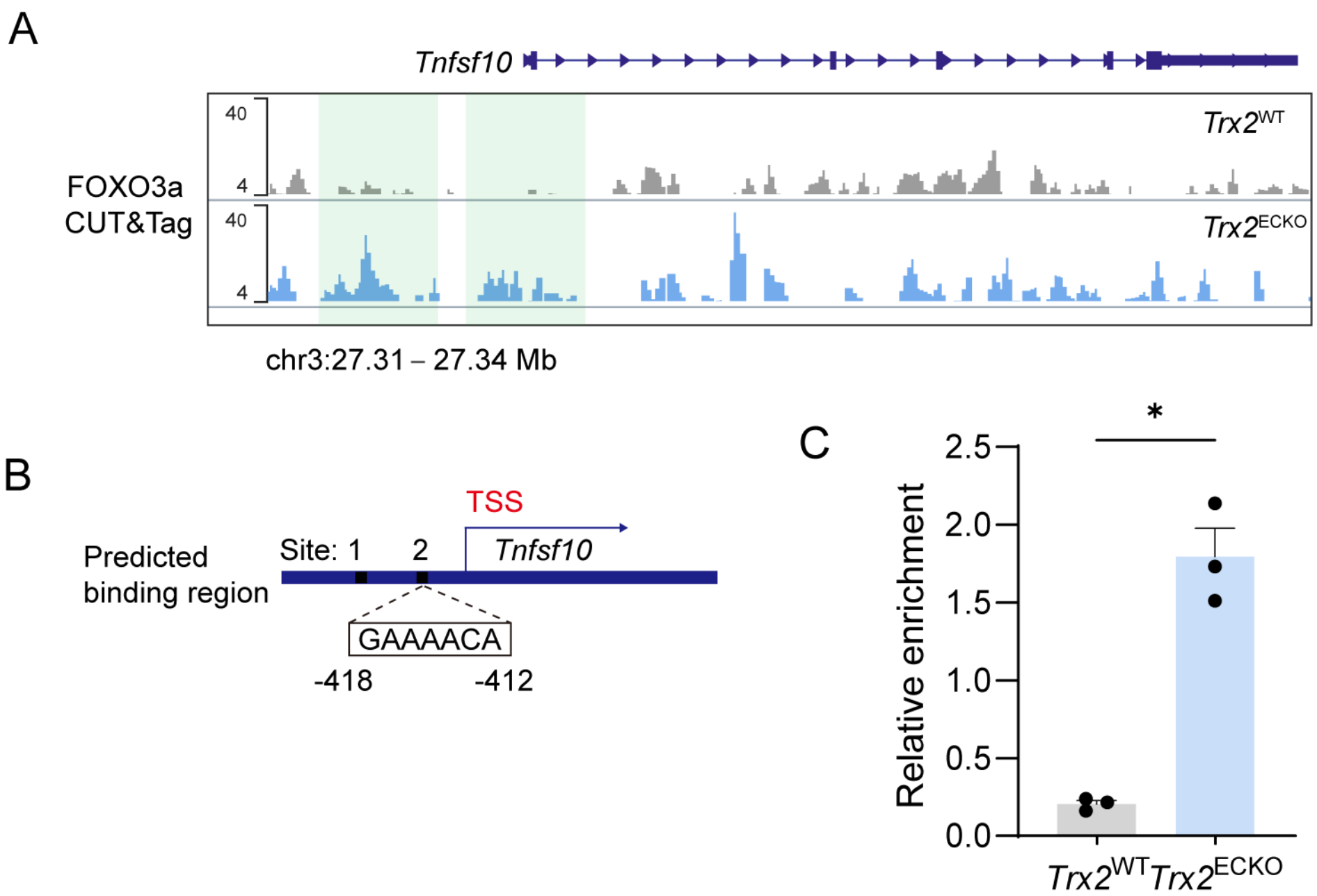

Figure S5. Endothelial TRX2 deficiency enhances FOXO3a occupancy at the *Tnfsf10* promoter. A) Genome-browser tracks showing FOXO3a CUT&Tag signals across the *Tnfsf10* locus (chr3:27.31–27.34 Mb) in BVECs isolated from *Trx2*^{WT} and *Trx2*^{ECKO} mice. Shaded regions highlight differential FOXO3a enrichment. B) Schematic representation of two predicted FOXO3a-binding sites within the proximal promoter of *Tnfsf10*. Site 2 contains the predicted FOXO3a-binding motif GAAAACA, located from −418 to −412 bp relative to the transcription start site (TSS). C) Targeted CUT&Tag-qPCR analysis of FOXO3a enrichment at site 2 of the *Tnfsf10* promoter in *Trx2*^{WT} and *Trx2*^{ECKO} BVECs (n = 3). Data are presented as means ± SEM. Statistical significance was determined by a two-tailed unpaired Student's t test. *P < 0.05.

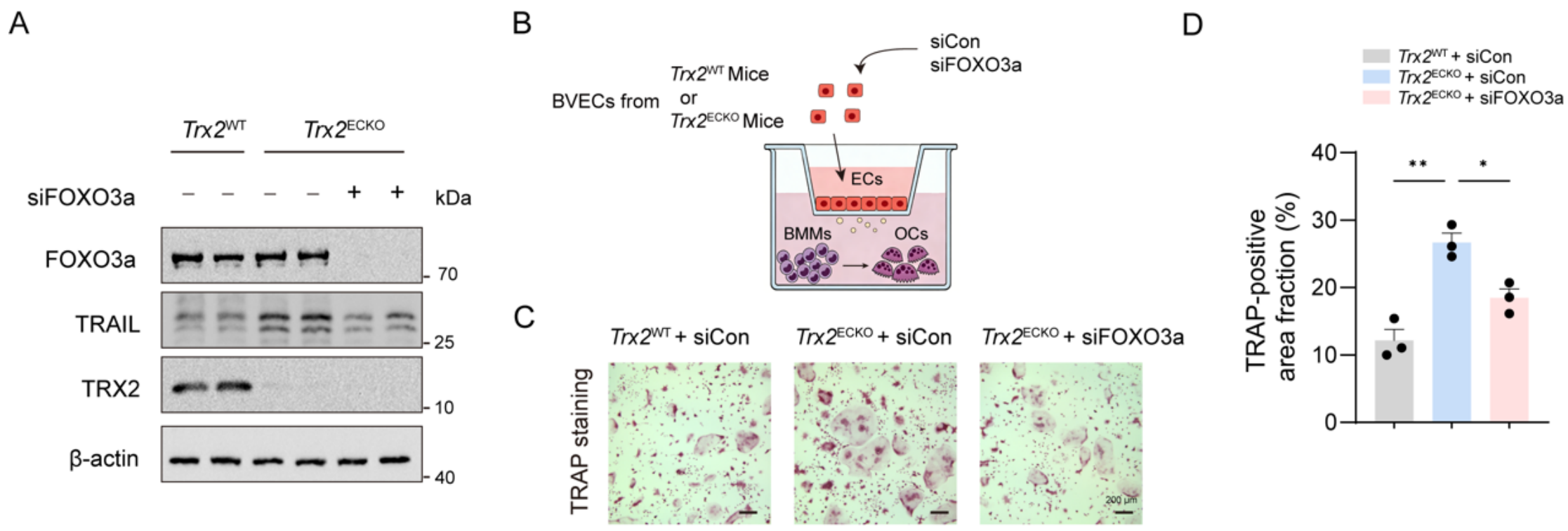

Figure S6. FOXO3a silencing attenuates endothelial TRX2 deficiency-induced TRAIL expression and osteoclastogenesis. A) Representative immunoblots of FOXO3a, TRAIL, and TRX2 in BVECs isolated from *Trx2*^{WT} and *Trx2*^{ECKO} mice and transfected with control siRNA (siCon) or siFOXO3a. B) Schematic illustration of co-culture system. BVECs subjected to the indicated treatments were cultured in the upper chamber, whereas bone marrow-derived macrophages (BMMs) were cultured in the lower chamber and induced to differentiate into osteoclasts. C,D) Representative images of TRAP staining (C) and quantification of the TRAP-positive area fraction (D) in the indicated groups (n = 3). Scale bars, 200 μm. Source data for the unedited raw immunoblots shown in Figure S10. Quantitative data are presented as means ± SEM. Statistical significance was determined by one-way ANOVA followed by Tukey's multiple-comparisons test. *$P < 0.05$, **$P < 0.01$.

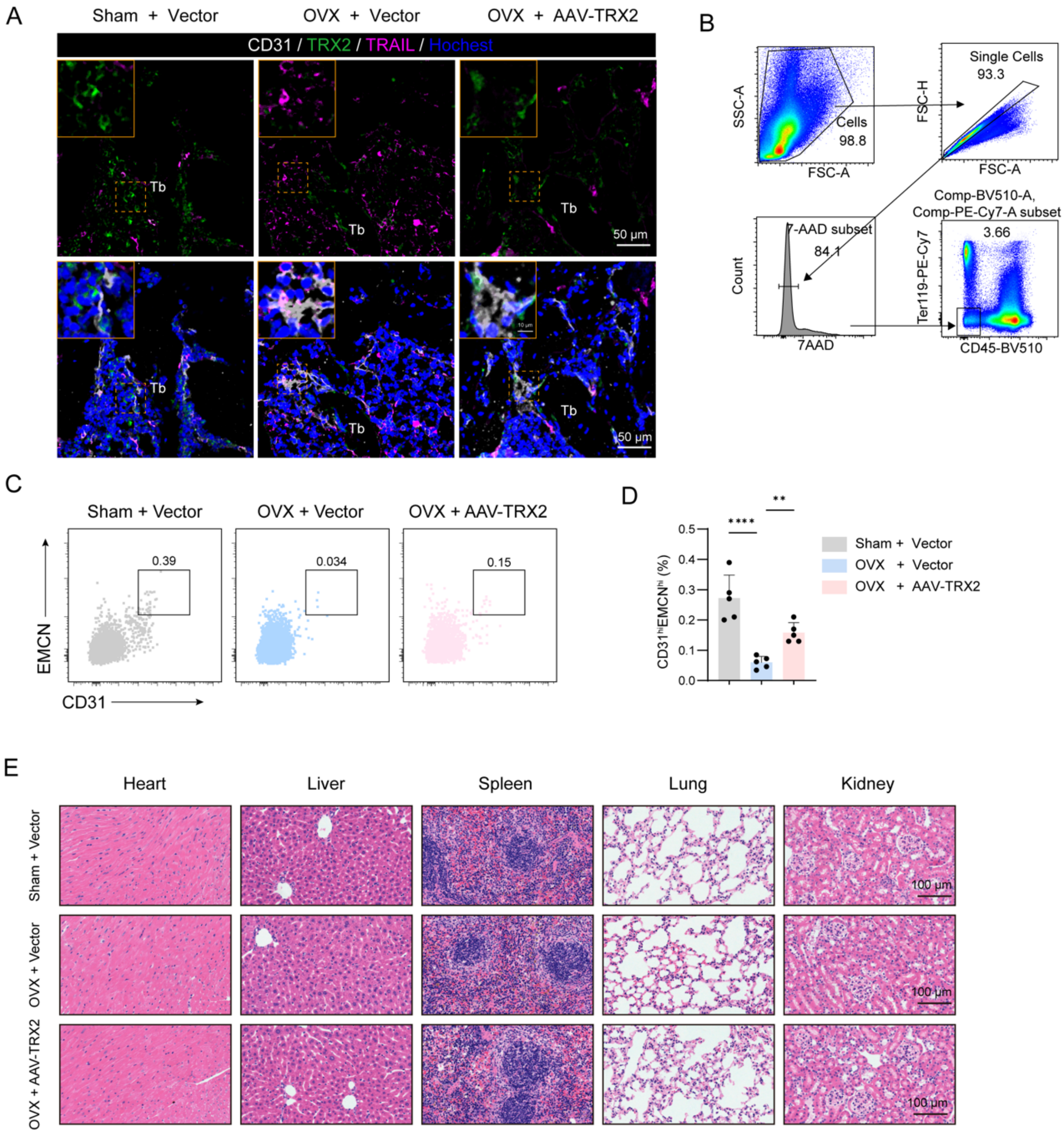

Figure S7. TRX2 restoration suppresses endothelial TRAIL expression, preserves type H vessels, and shows no overt systemic toxicity in vivo. A) Representative immunofluorescence images of CD31 (white), TRX2 (green), TRAIL (magenta), and Hoechst-stained nuclei (blue) in distal femurs from mice in the Sham + Vector, ovariectomy (OVX) + Vector, and OVX + TRX2–adeno-associated virus (AAV-TRX2) groups (n = 5). Boxed regions are shown at higher magnification in the insets. Scale bars, 50 μm. B) Representative flow cytometry gating strategy used to identify live 7-aminoactinomycin D–negative (7-AAD$^-$), lineage-negative CD45$^-$TER119$^-$ bone marrow cells. C) Representative flow cytometry plots showing type H endothelial cells, defined

as CD45⁻TER119⁻CD31^{hi}EMCN^{hi} cells, across the indicated treatment groups (n = 5). D) Quantification of the percentage of CD31^{hi}EMCN^{hi} type H endothelial cells. E) Representative hematoxylin and eosin (H&E)–stained sections of major organs, including heart, liver, spleen, lung, and kidney, showing no overt histopathological abnormalities after AAV-TRX2 administration. Scale bars, 100 μm. All quantitative data are presented as means ± SEM. Statistical significance was determined by one-way analysis of variance (ANOVA) with Tukey's multiple-comparisons test. **P < 0.01, ****P < 0.0001.

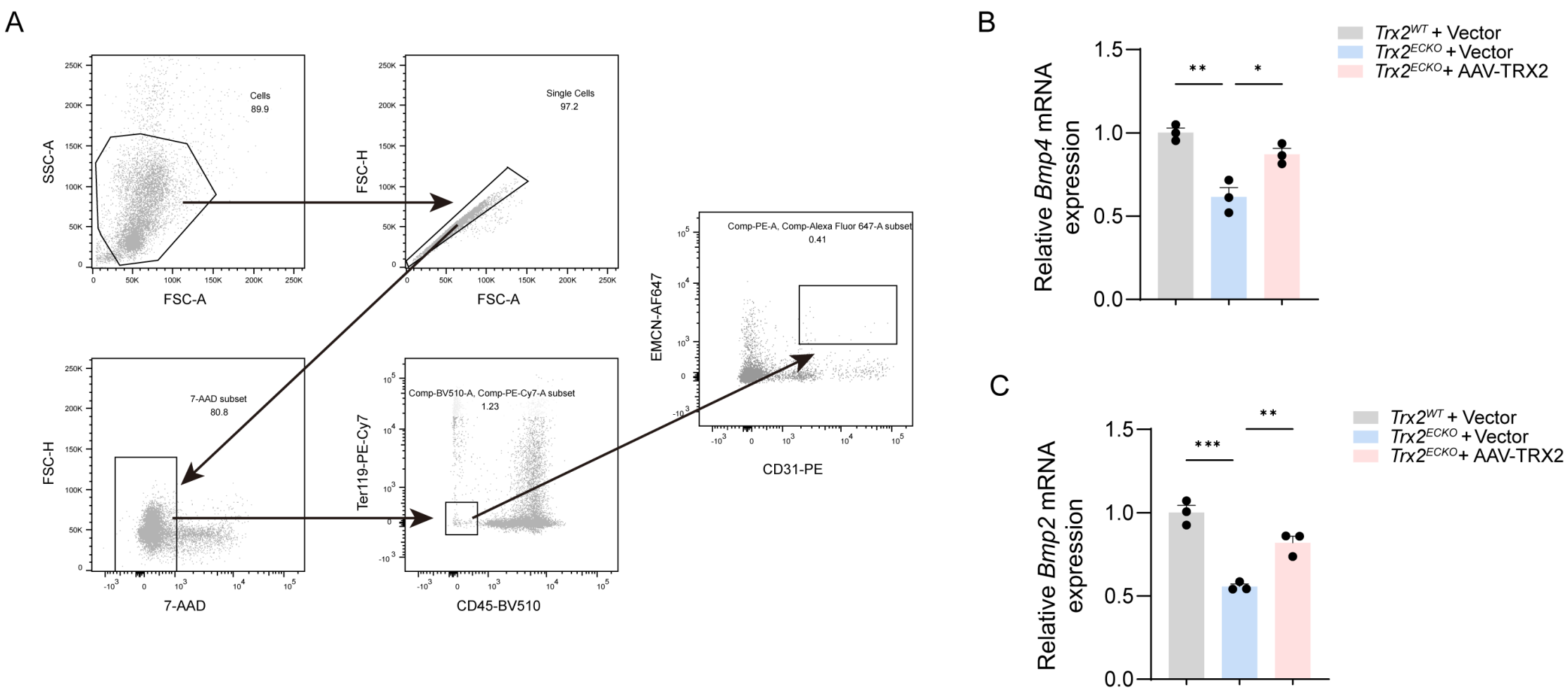

Figure S8. Endothelial TRX2 restoration partially restores pro-osteogenic angiocrine factor expression in type H endothelial cells. A) Representative flow cytometry gating strategy used to identify viable 7-aminoactinomycin D–negative (7-AAD⁻), CD45⁻TER119⁻ cells, followed by isolation of the CD31^{hi}EMCN^{hi} type H endothelial cells. B,C) Quantitative RT-PCR analysis of relative *Bmp4* (B) and *Bmp2* (C) mRNA expression in type H endothelial cells isolated from *Trx2*^{WT} + Vector, *Trx2*^{ECKO} + Vector, and *Trx2*^{ECKO} + AAV-TRX2 mice (n = 3). Data are presented as means ± SEM. Statistical significance was determined by one-way analysis of variance followed by Tukey's multiple-comparisons test. *P < 0.05, **P < 0.01, ***P < 0.001.

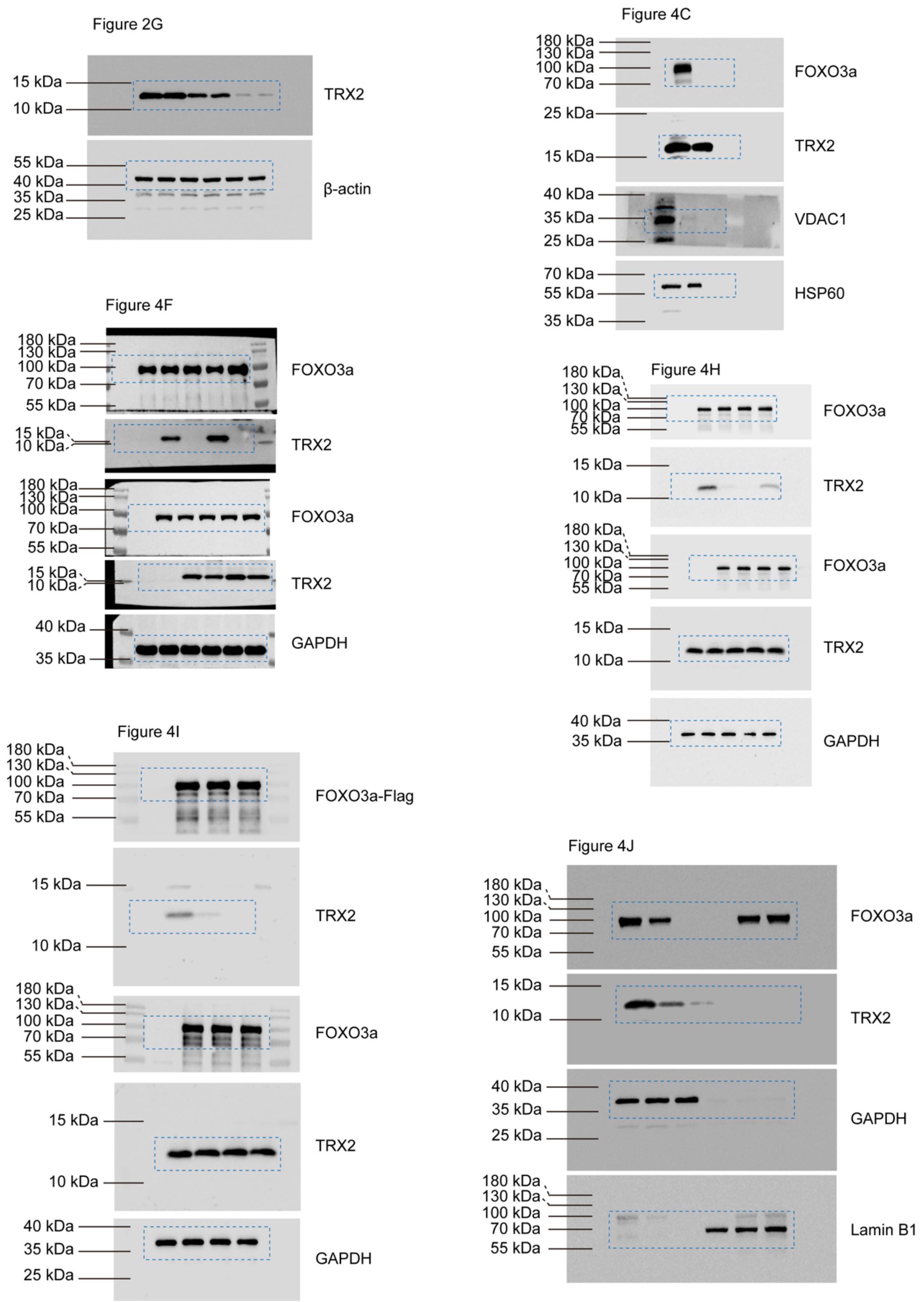

Figure S9. Source data for the unedited raw immunoblots shown in Figures 2 and 4.

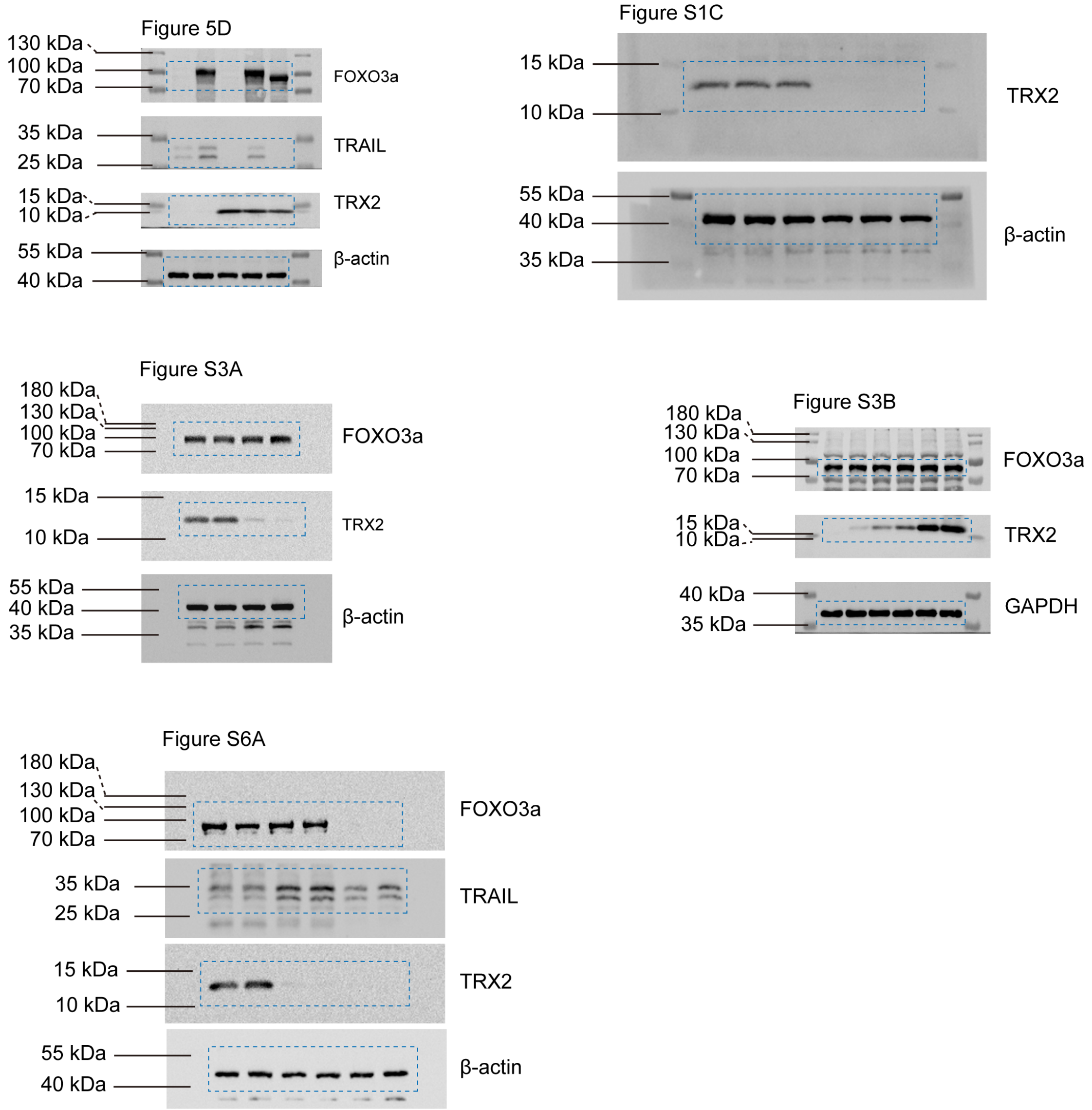

Figure S10. Source data for the unedited raw immunoblots shown in Figure 5, Figures S1, S3, and S6.

Table S1. Plasmids constructed in this study.

Plasmid Name	Insert Gene/Variant	Modification/Mutation Details
pcDNA3.1-hTRX2(WT)-HA	H. sapiens *TRX2*(WT)	Full-length wild-type sequence
pcDNA3.1-hTRX2(C90S)-HA	H. sapiens *TRX2*(C90S)	Point mutation at Cys90 to Ser
pcDNA3.1-hTRX2(C93S)-HA	H. sapiens *TRX2*(C93S)	Point mutation at Cys93 to Ser
pcDNA3.1-hTRX2(C90/93S)-HA	H. sapiens *TRX2*(C90/93S)	Double point mutations at Cys90 and Cys93 to Ser
pcDNA3.1-hFOXO3a(WT)-Flag	H. sapiens *FOXO3a*(WT)	Full-length wild-type sequence
pcDNA3.1-hFOXO3a(C31S)-Flag	H. sapiens *FOXO3a*(C31S)	Point mutation at Cys31 to Ser
pcDNA3.1-hFOXO3a(ΔCys)-Flag	H. sapiens *FOXO3a*(ΔCys)	Multi-site mutations (all targeted Cys to Ser)
pcDNA3.1-hFOXO3a(ΔCys-C31)-Flag	H. sapiens *FOXO3a(*ΔCys-C31)	Multi-site mutations with Cys31 left intact
pcDNA3.1-hTRX2(WT)-RFP	H. sapiens *TRX2*(WT)	Full-length wild-type sequence
pcDNA3.1-hFOXO3a(WT)-GFP	H. sapiens *FOXO3a*(WT)	Full-length wild-type sequence
pcDNA3.1-hFOXO3a-ΔNLS-Flag	H. sapiens *FOXO3a*(ΔNLS)	Deletion of the nuclear localization signal (NLS) region spanning amino acids 242–271 of human FOXO3a to abrogate nuclear translocation
pcDNA3.1-GFP-Flag	GFP-Flag	/
pLenti-GFP-Flag	GFP-Flag	/
pLenti-hTRX2(WT)-Flag	H. sapiens *TRX2*(WT)	Full-length wild-type sequence

pLenti-hFOXO3a(WT)-Flag	H. sapiens *FOXO3a*(WT)	Full-length wild-type sequence
pLKO.1-shTRX2/Trx2-RFP	H. sapiens/M. musculus *TRX2/Trx2* shRNA; target sequence: 5′-GCCTTCCTGAAGAAGCTGATT-3′	Targeting a conserved region of human *TRX2* and mouse *Trx2* for knockdown

Table S2. Primer and siRNA sequences used in this study.

Name	Sequences (5' -> 3')
siTRX2	Target sequence: GGATGGACCTGACTTTCAA
siFOXO3a	Target sequence: GAGCTCTTGGTGGATCATC
Bmp2	Forward: TTGGACACCAGGTTAGTGAATCA<br>Reverse: TCTCCTCTAAATGGGCCACTT
Bmp4	Forward: ATTCCTGGTAACCGAATGCTG<br>Reverse: CCGGTCTCAGGTATCAAACTAGC
Actb	Forward: GTGACGTTGACATCCGTAAAGA<br>Reverse: GCCGGACTCATCGTACTCC
Tnfsf10 CutTag-qPCR	Forward: AAACTCCCCCCAGATAAAAACA<br>Reverse: TGTGGACAGAGGGATAAACTGT
